# Unveiling the Cuproptosis in Colitis and Colitis-Related Carcinogenesis: A Multifaceted Player and Immune Moderator

**DOI:** 10.34133/research.0698

**Published:** 2025-05-14

**Authors:** Jingwen Liu, Hairuo Huang, Xiaojie Zhang, Yang Shen, DeMing Jiang, Shurong Hu, Shuyan Li, Zelin Yan, Wen Hu, Jinhua Luo, Haibo Yao, Yan Chen, Bufu Tang

**Affiliations:** ^1^ Department of Gastroenterology, the Second Affiliated Hospital, Zhejiang University School of Medicine, Hangzhou 310009, China.; ^2^ China Medical University, Shenyang 110122, China.; ^3^ The Fifth Affiliated Hospital of Wenzhou Medical University, Lishui 323000, China.; ^4^ Department of Radiation Oncology, Zhongshan Hospital, Fudan University, Shanghai 200000, China.; ^5^ Biosensor National Special Laboratory, Key Laboratory for Biomedical Engineering of Education Ministry, Department of Biomedical Engineering, Zhejiang University, Hangzhou 310007, China.; ^6^ Department of Nursing, the Second Affiliated Hospital, Zhejiang University School of Medicine, Hangzhou 310009, China.; ^7^ Department of Gastroenterology, the First Affiliated Hospital of Zhejiang Chinese Medical University, Zhejiang Provincial Key Laboratory of Gastrointestinal Diseases Pathophysiology, Hangzhou 310006, China.; ^8^ Department of Gastrointestinal and Pancreatic Surgery, Zhejiang Provincial People’s Hospital, Key Laboratory of Gastroenterology of Zhejiang Province, Hangzhou 310014, China.; ^9^ Department of Interventional Radiology, Zhongshan Hospital, Fudan University, Shanghai 200000, China.

## Abstract

Cuproptosis represents a novel mechanism of cellular demise characterized by the intracellular buildup of copper ions. Unlike other cell death mechanisms, its distinct process has drawn considerable interest for its promising applications in managing inflammatory bowel disease (IBD) and colorectal cancer (CRC). Emerging evidence indicates that copper metabolism and cuproptosis may exert dual regulatory effects within pathological cellular environments, specifically modulating oxidative stress responses, metabolic reprogramming, and immunotherapeutic efficacy. An appropriate level of copper may promote disease progression and exert synergistic effects, but exceeding a certain threshold, copper can inhibit disease development by inducing cuproptosis in pathological cells. This makes abnormal copper levels a potential new therapeutic target for IBD and CRC. This review emphasizes the dual function of copper metabolism and cuproptosis in the progression of IBD and CRC, while also exploring the potential application of copper-based therapies in disease treatment. The analysis further delineates the modulatory influence of tumor immune microenvironment on cuproptosis dynamics, while establishing the therapeutic potential of cuproptosis-targeted strategies in circumventing resistance to both conventional chemotherapeutic agents and emerging immunotherapies. This provides new research directions for the development of future cuproptosis inducers. Finally, this article discusses the latest advances in potential molecular targets of cuproptosis and their related genes in the treatment of IBD and CRC, highlighting future research priorities and unresolved issues.

## Introduction

Inflammatory bowel disease (IBD) and colorectal cancer (CRC) are worldwide health concerns, characterized by increasing prevalence and death rates. making them major threats to human health [1,2]. In defending against pathological cells, both involve various cells from innate and adaptive immunity [3]. IBD is fundamentally mediated by immune surveillance mechanisms that detect commensal microbiota and counteract proinflammatory mediators through coordinated mucosal barrier defenses, whereas CRC predominantly engages T cell-mediated immunosurveillance to detect and neutralize aberrant neoplastic cell proliferation through tumor-associated antigen recognition systems [4,5]. IBD represents a collection of persistent and recurrent inflammatory disorders that target the gastrointestinal system. Despite extensive research, the underlying mechanisms driving its development remain largely elusive [6,7]. It is widely accepted that the pathogenesis of the disease arises from a multifaceted interaction among genetic predisposition, environmental triggers, microbial influences, and immune system dysregulation. While these elements are crucial to disease development, none of them alone is adequate to initiate the disease, with the immune microenvironment serving as a key contributor [8,9]. On the other hand, CRC stands as a prevalent form of cancer affecting the digestive system. Early diagnosis and treatment remain challenging, and the prognosis is poor, with increasing incidence and mortality rates. CRC accounts for approximately 10% of global cancer incidence and mortality, ranking as the second most prevalent etiology of cancer-related deaths worldwide [10]. Its pathogenesis is similar to that of most cancers, starting with abnormal crypts and gradually progressing to precancerous lesions (polyps), eventually developing into malignant tumors over 10 to 15 years. Current therapeutic strategies encompass endoscopic or surgical resection for localized lesions, neoadjuvant radiotherapy to achieve tumor downstaging, and systemic therapeutic regimens incorporating molecular-targeted agents and immune checkpoint inhibitors, reflecting contemporary advances in precision medicine [11]. Chronic inflammation has long been associated with the development of cancer. Patients with IBD are at a higher risk of developing CRC, which represents a severe complication of chronic inflammation. This risk correlates with disease duration, extent of inflammation, and cumulative inflammatory burden [12]. Therefore, elucidating the pathogenesis of IBD and the molecular mechanisms underlying inflammatory microenvironment alterations is crucial for both IBD treatment and the prevention of IBD-associated CRC. With societal development, changes in lifestyle and dietary habits have correspondingly increased the rate of IBD and CRC. The occurrence of IBD ranges from 7% to 14%, while CRC incidence is approximately 10%, posing marked health risks to individuals, families, and society [10,13]. This urgent public health issue necessitates the advancement of innovative diagnostic approaches and treatment modalities, including targeted drugs.

Copper (Cu) plays an indispensable role in maintaining essential biological processes and physiological homeostasis, while its homeostatic imbalance can lead to cell apoptosis and various diseases. For instance, excessive Cu levels result in Wilson’s disease [14], whereas Cu deficiency causes Menkes disease [15]. We have systematically categorized the differential effects of varying copper levels in vivo to elucidate how specific concentration thresholds may trigger tumorigenic responses (Table 1).

**Table 1.** The major influence of different copper concentration

Copper concentration	Major influence	References
220 mg/kg	Nuclear deformation and shrinkage were observed in broiler chickens, along with partial disintegration of mitochondrial cristae	[188]
330 mg/kg	Vacuolar degeneration, fragmentation, and disappearance of mitochondrial cristae	[188]
20 mg/kg	ROS production was significantly increased, potentially triggering inflammation	[189]
1.252 (1.124–1.536) μg/ml	Induce breast cancer	[190]
131 ± 20 μg/dl ± SD	Induce breast cancer	[191]
125 ± 20.2 μg/dl ± SD	Induce lung cancer	[192]
124 ± 8.3 μg/dl ± SD	Induce prostate cancer	[193]
165 ± 33.9 μg/dl ± SD	Induce colorectal cancer	[194]
9.31 μmol/l	Copper content is high in gastric cancer and related to tumor progression	[195]

Research on cell death induced by Cu has been conducted for many years, and it was only in 2022 that Tsvetkov uncovered a novel type of regulated cell death resulting from Cu, termed “cuproptosis” [16]. Excess intracellular Cu targets lipoacylated proteins within the tricarboxylic acid (TCA) cycle, leading to their accumulation and triggering cell death. This discovery has opened new therapeutic avenues for treating IBD and CRC [17]. Cuproptosis exerts complex bidirectional regulatory effects on IBD and CRC. On the one hand, excessive Cu promotes inflammation and tumor progression through mechanisms such as enhanced cell proliferation, induction of drug resistance, stimulation of angiogenesis and metastasis, and increased reactive oxygen species (ROS) production [18–20]. On the other hand, disease suppression can also be accomplished through the use of copper chelators, which suppress angiogenesis while controlling cancer progression and metastasis, targeting cuproptosis to promote tumor cell death, and utilizing copper ion carriers for immunoregulation [21–23]. This review systematically delineates the ambivalent regulatory mechanisms of copper homeostasis and cuproptosis in the pathophysiology of IBD and CRC, while elucidating the therapeutic efficacy of copper-modulating agents through comprehensive analysis of their molecular targets and clinical translational potential. Additionally, we elucidate the impact of the immune microenvironment on cuproptosis and highlight the possibility of modulating cuproptosis as a therapeutic approach to overcome resistance to chemotherapy and immunotherapy, providing new insights for the advancement of next-generation pharmacological agents that induce cuproptosis. Finally, we discuss the latest progress in potential molecular targets of cuproptosis and associated genes in managing IBD and CRC, highlighting the key areas of future research and the pressing issues that need to be addressed.

## Cu Metabolism and Cuproptosis

### Cu metabolism

**Fig. 1.**
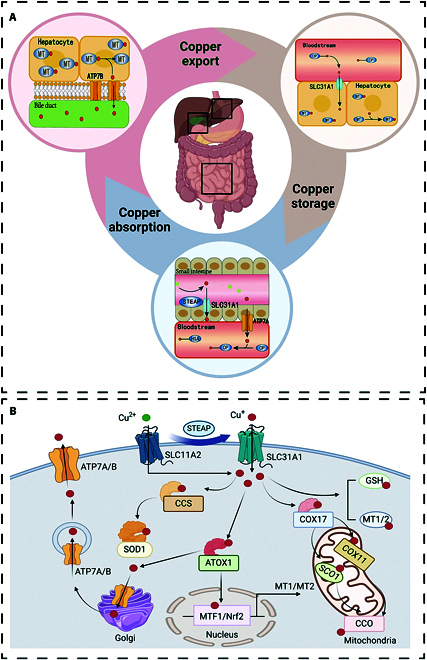
Schematic representation of copper homeostasis processes. (A) Copper uptake, storage, and distribution. Cu absorption primarily occurs within the duodenal and small intestinal regions, where the epithelial cells of the intestine absorb Cu mainly via the SLC31A1. After absorption, Cu in its divalent form is transported into circulation through ATP7A. Within the bloodstream, Cu binds to transport proteins and is conveyed to the liver via the portal circulation. The liver serves as the primary organ for Cu storage and systemic Cu regulation. Within hepatocytes, Cu is stored by binding to metallothioneins to prevent oxidative stress caused by free Cu ions. Excess Cu is excreted into the bile and subsequently eliminated through feces, a process mediated by the ATP7B transporter, which is critical for maintaining Cu homeostasis. A minor portion of Cu is also excreted via sweat glands, urine, and other pathways. (B) Intracellular Cu handling. STEAP (six-transmembrane epithelial antigen of the prostate) converts Cu^2+^ to Cu^+^. SLC31A1 aids in the transfer of Cu^+^ into the cytoplasm, while ATP7A/B exports Cu^+^ out of the cell. CCS, a Cu chaperone protein, contains Cu-binding sequences and delivers Cu to SOD1. Within the cell, copper interacts with the chaperone protein Atox1, creating a stable complex that facilitates secure copper ion transport. Subsequently, this complex interacts with the copper-transporting ATPases ATP7A or ATP7B within the Golgi network, forming a functional complex.

Cu constitutes an indispensable trace element crucial for maintaining optimal physiological functions in humans, participating in various signaling pathways and closely associated with inflammatory factors and tumor-related molecules [24]. The principal dietary sources of Cu comprise organ meats, meat, and shellfish, with a recommended daily intake of 0.8 to 2.4 mg to maintain systemic Cu homeostasis [25]. Dietary Cu absorption mainly takes place in the initial segment of the small bowel and its adjoining regions and depends on Cu transport protein 1 (CTR1), also referred to as solute carrier family 31 member 1 (SLC31A1), and excessive absorption of Cu ions via CTR1 can lead to cuproptosis. Once absorbed, Cu is transferred into circulation through ATPase copper transporting alpha (ATP7A) and transported to the hepatic region via the portal circulation, where it is primarily stored. Within hepatocytes, Cu is predominantly sequestered by metallothionein 1 and 2 for storage [26–28]. Subsequently, Cu is released back into the bloodstream through ATPase copper transporting beta (ATP7B). In this phase, it binds once again to soluble chaperone proteins, which play a crucial role in safely transporting copper to various specific tissues and organs where it is needed for essential biological functions [29]. In target tissues, Cu modulates diverse physiological processes, encompassing immune regulation, redox homeostasis maintenance, and intestinal microbiota modulation. Excess Cu is excreted via feces after binding to amino acids in bile, with a small portion excreted through the intestinal epithelium [30] (Fig. 1).

### Cuproptosis

**Fig. 2.**
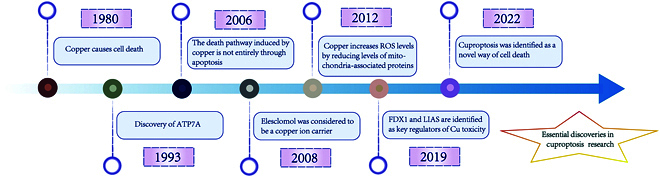
This figure provides a detailed overview of the primary discoveries and significant insights gained from the study of the cuproptosis process. It highlights the key mechanisms, pathways, and molecular interactions involved in this cellular demise mechanism, offering a comprehensive understanding of its biological significance and implications.

First proposed by Tsvetkov and colleagues in 2022, the term “cuproptosis” describes a newly identified pathway through which copper triggers cellular demise (Fig. 2). This process distinguishes itself from established cell death modalities, including apoptosis and necrosis, but more closely resembles ferroptosis. Tsvetkov et al. demonstrated that copper ionophore-induced cell death occurs independently of classical apoptotic markers, as evidenced by the absence of caspase-3 cleavage or activation. Notably, genetic ablation of key apoptotic effectors BAX and BAK1, or pharmacological inhibition of caspases using pan-caspase inhibitors, failed to prevent copper ionophore-mediated cell death, clearly distinguishing this process from apoptosis. Furthermore, neither necroptosis inhibitors nor ferroptosis inhibitors could attenuate copper-induced cell death [16]. Cuproptosis, on the other hand, is characterized by a unique mechanism and cannot be reversed or rescued using traditional cell death inhibitors (e.g., apoptosis inhibitors or ferroptosis inhibitors). Consequently, the application of copper chelators can effectively remove excess copper ions, thereby preventing copper-induced cellular damage and rescuing the cells [16].

**Fig. 3.**
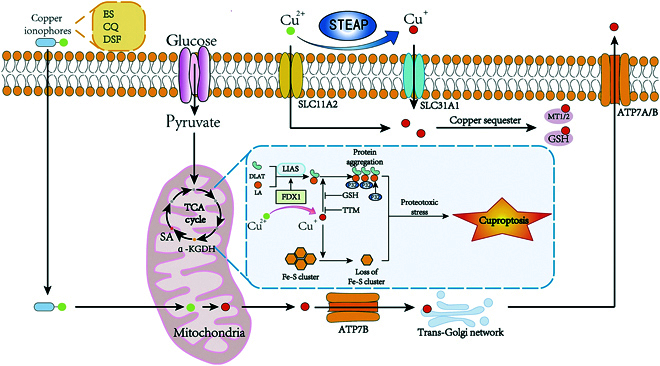
Schematic of the cuproptosis mechanism. This figure summarizes the mechanisms and processes involved in Cu-induced cell death. SLC31A1, in conjunction with copper ion carriers like ES and DSF, facilitates the uptake of copper ions, resulting in a substantial rise in cellular copper concentrations. These transporters move Cu^2+^ into mitochondria, where FDX1 reduces it to Cu^+^. The mitochondrial matrix houses multiple enzymes, such as LIAS and DLAT, that participate in the TCA cycle. FDX1 plays a role in electron transfer during metabolism, directly or indirectly regulating the proper functioning of the TCA cycle alongside copper ions. Copper ions directly interact with lipoacylated DLAT in the mitochondrial TCA cycle, causing the clumping of lipoylated proteins, the depletion of Fe-S clusters, and the misfolding and buildup of proteins. These events lead to mitochondrial dysfunction, such as disruptions in oxidative phosphorylation and overabundant buildup of ROS, ultimately resulting in cuproptosis. Additionally, ATP7A/B can expel excess copper from cells or convey it to the Golgi network for integration into copper-requiring enzymes.

Research has demonstrated that Cu ionophores, like disulfiram (DSF) [31] and elesclomol (ES) [32], promote Cu overload within cells, particularly in mitochondria [33]. Excessive copper levels directly interact with lipoacylated elements in the TCA cycle, triggering the buildup of lipoacylated proteins and the reduction of Fe-S cluster proteins, and protein misfolding. This results in mitochondrial dysfunction, such as disrupted oxidative phosphorylation and the excessive accumulation of ROS [16]. The specific mechanism involves the reduction of Cu^2+^ to Cu^+^ by ferredoxin 1 (FDX1) in mitochondria. FDX1 is recognized as a key upstream regulator of the lipoic acid (LA) pathway. Governed by FDX1, lipoic acid synthetase (LIAS) facilitates the attachment of LA moieties to dihydrolipoamide *S*-acetyltransferase (DLAT). Cu ions form direct coordination bonds with the LA moiety of lipoacylated DLAT through disulfide linkages, inducing DLAT oligomerization and subsequent proteotoxic stress [16]. As mitochondrial dysfunction progresses and ROS levels rise, intracellular stress response pathways are activated, inducing cell death via multiple pathways characteristic of cuproptosis [34] (Fig. 3). Given that the intestine serves as the main site for copper absorption, dysregulated Cu metabolism may induce cuproptosis in intestinal epithelial or immune cells, further exacerbating the loss of intestinal barrier function. This process may exacerbate intestinal inflammation and promote tumorigenesis, thereby establishing a mechanistic link between Cu-induced cytotoxicity and the pathogenesis of both inflammatory disorders and malignant transformations in the gastrointestinal tract.

The mechanisms of cuproptosis hold promising potential for clinical treatment across various diseases. The molecular components implicated in this process may be exploited to trigger cuproptosis in pathological cells, offering innovative therapeutic avenues for drug development (Table 2) [16,17,35–47]. In the course of cuproptosis induction, FDX1 serves as a key upstream regulator of the LA pathway, serving a pivotal role in controlling protein lipoylation. In CRC, reduced FDX1 expression is frequently linked to advanced stages of cancer and metastatic features, resulting in shorter overall survival [48,49]. Moreover, the dysregulation of Cu transport proteins, including ATP7A, ATP7B, and SLC31A1, can disrupt cellular function [24]. SLC31A1 regulates copper uptake into cells, whereas ATP7A and ATP7B regulate copper efflux, all of which is essential in Cu transport and homeostasis [50–52]. However, further investigation is required to elucidate the precise molecular mechanisms governing these transporters’ function, their associated genetic regulators, and their role in cuproptosis induction. This will be essential for translating the molecular mechanisms of cuproptosis into actionable therapeutic strategies for CRC and other diseases.

**Table 2.** Regulatory proteins and genes associated with cuproptosis

Genes/proteins	Impact on cuproptosis	Materials	Major functions	References
FDX1	Positive	OVISE, ABC1	FDX1 acts as a key regulator of protein lipoylation by reducing Cu^2+^ to Cu^+^. Genetic deletion of FDX1 inhibits cuproptosis triggered by elesclomol–Cu and disulfiram–Cu	[16,35,36]
LIAS	Positive	OVISE, ABC1	Genetic deletion of LIAS inhibits cuproptosis triggered by elesclomol–Cu and disulfiram–Cu	[16,17,37]
LIPT1	Positive	OVISE	Genetic deletion of LIPT1 inhibits cuproptosis triggered by elesclomol–Cu and disulfiram–Cu	[17,38,39]
DLAT	Positive	OVISE	Copper binds directly to lipoylated DLAT and promotes its oligomerization, causing proteotoxic stress. Deletion of DLAT reduces cell death caused by elesclomol–Cu and disulfiram–Cu	[16,17,38]
SLC31A1	Positive	OVISE, 4T1 tumor	Excessive activation of SLC31A1 increases intracellular copper accumulation	[40–42]
ATP7A/B	Negative	OVISE	Knocking out ATP7A/B promotes the accumulation of Cu within cells	[42–44]
GLS	Negative	OVISE, 4T1 tumor	Genetic deletion of GLS inhibits cuproptosis triggered by elesclomol–Cu and disulfiram–Cu	[16,17]
DLD	Positive	OVISE	Genetic deletion of DLD inhibits cuproptosis triggered by elesclomol–Cu and disulfiram–Cu	[16,37,45]
PDHA1	Positive	OVISE	Genetic deletion of PDHA1 inhibits cuproptosis triggered by elesclomol–Cu and disulfiram–Cu	[16,17]
PDHB	Positive	OVISE	Genetic deletion of PDHB inhibits cuproptosis triggered by elesclomol–Cu and disulfiram–Cu	[17,46]
MTF1	Negative	OVISE	Genetic deletion of MTF1 inhibits cuproptosis triggered by elesclomol–Cu and disulfiram–Cu	[16,17,47]
CDKN2A	Negative	OVISE	Genetic deletion of CDKN2A inhibits cuproptosis triggered by elesclomol–Cu and disulfiram–Cu	[16,17,38]

## Interaction between Cuproptosis and the Intestinal Immune Microenvironment

### Immune microenvironment

The tumor microenvironment (TME) is characterized as the local cellular environment surrounding tumors or cancer stem cells [53]. The neoplastic milieu consists of various immunocytes, lymphocytes, extracellular matrix (ECM), vascular networks, and inflammatory cell populations [54]. The immune microenvironment, as the core component of the TME, plays an essential role in maintaining body health, participating in immune responses, and influencing tumor progression. Current evidence demonstrates that both immune cell populations and stromal components play critical roles in the activation and aggregation processes, and reprogramming of the extracellular matrix, and these changes result directly from the intricate crosstalk between tumor cells and the TME [55]. Evaluation of the immune microenvironment provides significant prognostic value and represents a robust adjunct to conventional histopathological and molecular biomarker analysis. This integrated evaluation approach allows for more accurate predictions of patient responses to immunotherapy, providing more comprehensive evidence for personalized treatment strategies [56] (Table 3).

**Table 3.** Copper-dependent biomarkers associated with cuproptosis in cancer

Biomarkers	Cancer type	Major functions	References
AURKA	Head and neck squamous cell carcinoma	Up-regulated in TP53-mutant/HPV-negative HNSCC and correlated with poor prognosis	[196]
EREG	Glioblastoma	EREG modulates VEGF/CD99 crosstalk in GBM and supports chemo/immunotherapy	[197]
CRGs	Head and neck squamous cell carcinoma	Serving as a molecular prognostic marker	[198]
FDX1	Glioma	Serving as a new type of glioma patient immunotherapy markers	[199]
LIPT1	Skin cutaneous melanoma	Serving as an independent reliable prognostic marker in multivariate analysis	[200]
CRGs	Hepatocellular carcinoma	Serving as a novel candidate prognostic biomarker	[201]
DARS2	Hepatocellular carcinoma	Serving as a potentially reliable prognostic biomarker	[202]
METTL3	Oesophageal carcinoma	Serving as a potential biomarker associated with tumorigenesis and progression	[203]
DLAT	Colorectal cancer	Serving as a predictive and immunotherapy biomarker	[204]
DLAT	Pancreatic adenocarcinoma	Serving as a promising prognostic and immune biomarker	[205]
IRG	Lung adenocarcinoma	Serving as a suitable biomarker for immunotherapy	[206]
CRIGs	Gastric cancer	Serving as an immune biomarker	[207]
LIAS	Cancer	Serving as a potential prognostic biomarker	[208]
PPIC	Cutaneous melanoma	Serving as a promising predictive and therapeutic biomarker	[209]
CRGs	Hepatocellular carcinoma	Serving as a therapeutic biomarker targeting the tumor immune microenvironment and immune checkpoints	[210]
CRGs	Colon adenocarcinoma	Serving as a clinical biomarker	[211]
SLC31A1	Breast cancer, liver cancer	Serving as a candidate biomarker or therapeutic target for precision oncology	[212]
SLC31A1	Liver cancer	Serving as a poor prognostic markers for liver cancer	[213]
lncRNA	Breast, lung, liver, ovarian, pancreatic, and gastric cancers	Serving as a potential prognostic biomarker and therapeutic target in cuproptosis-mediated cancers	[214]

In recent years, Cu, as an essential element for the function of various immune cells, has been found to influence the balance of the immune system. Both excess and deficiency of Cu can disrupt immune homeostasis, promoting an immunocompromised state. For instance, cytokines in the immune microenvironment (e.g., tumor necrosis factor-α [TNF-α] and interleukin-10 [IL-10]) regulate the expression of Cu transport proteins, thereby affecting Cu uptake, excretion, and distribution within the body, ultimately altering Cu metabolism [34]. Understanding the interactions between Cu metabolism, cuproptosis, and the immune microenvironment not only sheds light on their roles in disease pathogenesis but also provides new research directions and treatment approaches for disease treatment.

### Induction of immune antitumor effects by cuproptosis

Immunogenic cell death (ICD) represents a unique type of controlled cell demise that has the capacity to elicit an adaptive immune response. A hallmark of ICD involves the secretion of DAMPs (damage-associated molecular patterns) [57]. DAMPs can be divided into 5 categories: nucleic acids, proteins, ions, glycans, and metabolites. These molecules facilitate antigen recognition and phagocytosis by binding to antigen-presenting cells, thereby enhancing apoptotic cell clearance. These antigens are then processed and displayed to T lymphocytes, initiating specific immune reactions [58]. Additionally, activation of DAMP-sensing receptors induces the secretion of inflammatory cytokines, promoting the attraction of immune cells and triggering inflammatory responses [59].

**Fig. 4.**
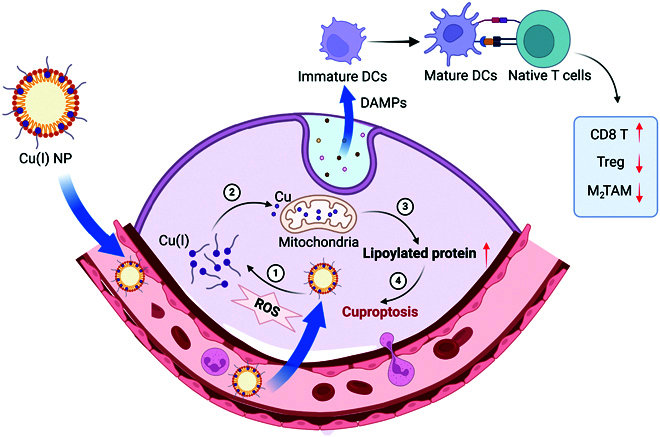
The mechanism by which Cu(I)NPs stimulate cancer cell death via cuproptosis and activate immune reactions is outlined below: Cu(I)NPs are transported through the bloodstream and specifically concentrate in the tumor location, where they release Cu(I). This release results in the production of ROS and the accumulation of lipoylated proteins, ultimately inducing cuproptosis. Simultaneously, the release of DAMPs promotes the maturation of immature DCs. These mature DCs further activate naïve T cells, enhancing the activity of CD8^+^ T cells while suppressing the population of regulatory T cells and M2-type tumor-associated macrophages (TAMs). This process strengthens the immune response against tumors and remodels the tumor immune microenvironment, contributing to improved immunotherapeutic outcomes.

Studies have demonstrated that Cu complex nanoparticles (Cu(I)NPs) capable of inducing cuproptosis can trigger ICD and activate adaptive immune responses. This process potently augments antitumor immunity and remodels the tumor immune microenvironment [58] (Fig. 4). Thus, specific activation of cuproptosis may help reactivate the TME and facilitate the eradication of cancer cells [60]. Currently, the immune regulatory molecules CTLA-4 (cytotoxic T-lymphocyte-associated protein 4) and PD-1 (programmed cell death protein 1) have been the focus of extensive investigation [61]. However, the efficacy of immune checkpoint inhibitors is often limited by the immunosuppressive TME. To overcome this obstacle, researchers have developed a novel therapeutic strategy based on cuproptosis. The copper ionophore celastrol not only effectively delivers copper ions but also depletes overexpressed glutathione (GSH) in cancer cells, thereby inducing a self-amplifying cuproptosis process within tumor cells. The celastrol–copper (Cel–Cu) coordination NPs formed by Cel and copper ions not only efficiently trigger cuproptosis but also induce ICD, promoting the reprogramming and maturation of the immune microenvironment. This combination strategy significantly suppresses tumor progression through the synergistic effects of cuproptosis and ICD, effectively overcoming drug resistance caused by the immunosuppressive microenvironment. Notably, the Cel–Cu NPs exhibit excellent tumor-targeted accumulation and favorable biosafety [62]. In addition, cuproptosis results in the exocytosis of antigens associated with tumors, which are detectable by the body’s defense mechanisms, thereby triggering the activation of immune responses. This mechanism potentiates antitumor immunity and synergizes with established immunotherapeutic regimens, augmenting their clinical efficacy. Therefore, cuproptosis activators could serve as a complementary strategy to existing immunotherapeutic drugs, providing stronger antitumor responses for cancer treatment [63].

### Role of cuproptosis in intestinal immunity

The intestinal barrier serves as the primary defense against pathogenic microbial invasion [64]. Its dysfunction is implicated in various diseases, including IBD, acute pancreatitis, and CRC [65–67]. Intestinal barrier homeostasis is co-regulated by commensal microbiota and the immune system. When a small number of commensal bacteria penetrate the mucus layer, they are captured by intestinal DCs and presented to T and B cells. This process stimulates B cells to produce bacteria-specific IgA antibodies. Concurrently, macrophages in the lamina propria are activated to perform phagocytic and secretory functions. Antimicrobial peptides (e.g., defensins) are secreted to eliminate commensal bacteria that approach epithelial cells [68,69]. Another mechanism maintaining intestinal homeostasis involves the aryl hydrocarbon receptor (AHR) pathway. AHR activation triggers IL-22 release, up-regulates IL-10 receptor expression, and enhances tight junction formation between intestinal epithelial cells, thereby reinforcing barrier integrity [70].

The intestinal immune response involves various cells from both innate and adaptive immunity, with STING (stimulator of interferon genes) pathway via cGAS (cyclic GMP-AMP synthase) emerging as a key innate immune signaling pathway [3,71,72]. Research has shown that cuproptosis can enhance cancer immunity through the cGAS-STING signaling pathway [73,74]. Tumor antigens released during cancer cell death are absorbed by dendritic cells (DCs) [75]. In DCs, cyclic GMP-AMP (cGAMP) activity is heightened, inducing a robust inflammatory response characterized by increased IL-2, TNF-α, and CXCL10/11 secretion, which collectively induce inflammation [74]. Additionally, Cu oxide NPs dissolve in macrophage lysosomes, inducing cuproptosis defined by mitochondrial dilation and indicators of oxidative stress [76]. DSF/Cu enhances M1 macrophage polarization while modulating glucose metabolic reprogramming via the mTOR (mechanistic target of rapamycin) pathway [77]. Moreover, the combination of DSF/Cu and CD47 inhibition demonstrates a synergistic therapeutic impact, facilitating DC maturation and significantly enhancing the cytotoxic function of CD8^+^ T cells, thus boosting the antitumor immune response [78]. Inducing cuproptosis in localized tumors significantly impacts the immune microenvironment, influencing tumor-infiltrating immune cells and improving immunotherapy outcomes. For example, IL-10, an anti-inflammatory cytokine secreted by intestinal DCs, functions to suppress inflammatory responses. Overactive inflammatory responses in macrophages during IBD may also serve as effective intervention points [79,80]. By leveraging the regulatory mechanisms of cuproptosis, new therapeutic strategies can be developed to alleviate intestinal diseases and modulate immune responses.

## Bidirectional Regulation of Cu in IBD

Cu plays a complex bidirectional role in IBD. Its metabolic balance and cuproptosis mechanisms function differently under varying pathological conditions. On one hand, as a crucial cofactor for key antioxidant enzymes, Cu effectively scavenges ROS and precisely modulates immune responses, thus maintaining gut barrier stability and reducing inflammatory responses [81]. On the other hand, Cu imbalance or overabundant accumulation can exacerbate oxidative stress, induce apoptosis or necrosis in intestinal epithelial cells, and weaken tight junction proteins, thereby promoting IBD progression [82]. Understanding the bidirectional regulation of Cu metabolism and cuproptosis in IBD offers valuable insights into its pathogenesis and provides theoretical grounds for developing targeted therapeutic strategies.

### Proinflammatory effects of Cu

#### Oxidative stress

ROS naturally arise as metabolic by-products during normal cellular physiological processes [83]. Within the inflammatory intestinal mucosal environment, the release of ROS and other inflammatory markers directly damages intestinal epithelial cells and disrupts the delicate pathways of proinflammatory reactive substances in immune cells [84]. Elevated ROS levels, combined with diminished antioxidant capacity, represent key pathogenic factors in IBD development [85].

Evidence increasingly suggests a close association between disrupted intracellular Cu metabolism and oxidative stress [86,87]. Excess copper, as a potent oxidant, induces ROS generation and elicits inflammatory responses. Studies have found that Cu ion carriers can induce ROS generation and activation of pro-apoptotic signaling pathways, containing the JNK (c-Jun N-terminal kinase) pathway and the MAPK (mitogen-activated protein kinase) pathway, and inhibit anti-apoptotic nuclear factor kappa B (NF-κB) signaling [88,89]. Additionally, cuproptosis specifically targets lipoacylated components associated with the TCA, resulting in the destabilization of Fe-S cluster proteins and consequent disruption of mitochondrial respiratory chain activity, which has been linked to IBD [90]. Impaired mitochondrial function induces a state of bioenergetic crisis, weakening epithelial barrier integrity by heightening prone to cuproptosis and decreasing the production of secretory barrier components, and impairing regeneration in response to damage [91–93]. To effectively manage IBD onset and progression, oxidative stress must be mitigated via antioxidant or anti-inflammatory strategies, such as Cu chelators [94], cuproptosis-related proteins like GSH [83], phenolic and polyphenolic compounds [95], and hormonal therapies [96,97].

#### Intestinal immune dysbiosis

Chronic intestinal inflammation involves dysbiosis of gut microbiota, compromised epithelial barriers, immune cell activation, and reduced immune tolerance to bacteria [98–100]. Under normal conditions, the gut microbiota and host immune system maintain a dynamic equilibrium to protect against pathogens and preserve intestinal homeostasis [101]. When small amounts of commensal bacteria penetrate the mucus layer, immune cells within the mucosal layer become activated to eliminate bacteria nearing epithelial cells by producing antimicrobial substances like defensins [69]. Activated macrophages are marked by increased mitochondrial Cu^2+^ levels, which catalyze NADH redox cycling and promote metabolic changes, triggering epigenetic modifications associated with inflammation [102]. Targeting mitochondrial Cu (Cu^2+^) has been shown to restore gut homeostasis by inhibiting the production of key metabolites necessary for initiating and sustaining inflammation [102]. Excessive copper intake induces oxidative damage, initiating cuproptosis in intestinal epithelial cells. This cascade disrupts gut barrier integrity, alters microbiota equilibrium, and establishes a proinflammatory feedback cycle [103–105]. Therefore, Cu ion levels are essential for immune cell metabolism and gut microbiota equilibrium, positioning Cu serving as a possible target for controlling uncontrolled inflammation [106].

### Anti-inflammatory effects of Cu

#### Regulation of the NF-κB pathway by COMMD1

The pathogenesis, progression, and treatment of IBD are complex processes, with Cu-related enzymes and proteins playing key roles. Critical examples include COMMD1 (copper metabolism MURR1 domain-containing protein 1), IκB (an NF-κB family member), and cytochrome c oxidase 17 (COX17). Strong evidence links NF-κB to the development of IBD. Within the inflamed intestinal mucosa of IBD patients, dysregulated expression of NF-κB precursors, NF-κB itself, and immune receptors stimulated by NF-κB (such as NOD2 [nucleotide-binding oligomerization domain-containing protein 2]) has been observed. Additionally, the regulation of downstream genes associated with NF-κB, such as interleukins IL-12 and IL-23, is also disrupted [107]. Persistent NF-κB activation drives mucosal inflammation and epithelial barrier disruption through up-regulation of proinflammatory cytokines and induction of intestinal epithelial cell apoptosis [108]. COMMD1 is a multifunctional protein involved in Cu excretion regulation in the liver, sodium uptake via epithelial sodium channels (ENaC), and NF-κB signaling modulation [109]. Growing evidence indicates that COMMD1 engages with the ubiquitin–proteasome pathway, influencing the degradation of NF-κB elements, ATP7B, and hypoxia-inducible factor-1α (HIF-1α) [110,111].

**Fig. 5.**
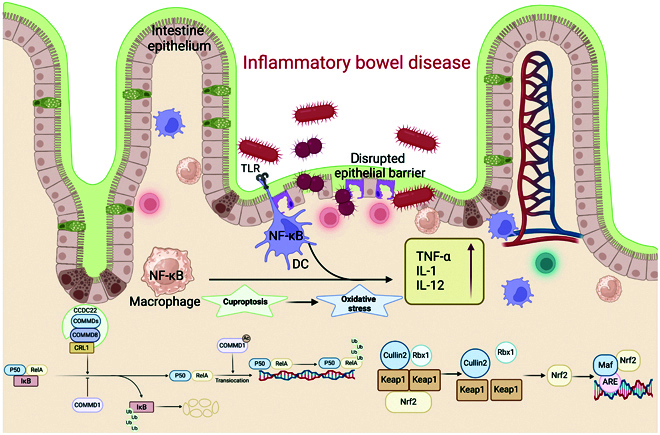
Schematic diagram of COMMD proteins and NF-κB pathway in regulating inflammation and oxidative stress caused by intestinal barrier dysfunction. In immune cells, oxidative stress induced by cuproptosis can promote the separation of the NF-κB/IκB complex and the Nrf2/Keap1 complex, thereby inducing the expression of proinflammatory and antioxidant genes. COMMD1 facilitates the ubiquitination of NF-κB by engaging with cullin2, further facilitating the dissociation of CAND1 within the cullin–ubiquitin ligase system. Through intricate molecular interactions, the crosstalk between these 2 pathways is vital in the progression of IBD.

Within the NF-κB signaling cascade, PAMPs (pathogen-associated molecular patterns)/DAMPs and inflammatory signaling molecules (e.g., TNF-α and IL-1) activate membrane receptors, leading to IκB phosphorylation by IκB kinase (IKK) and ensuing breakdown by the proteasome. This releases the RelA/p50 heterodimer, which translocates to the nucleus to modulate gene transcription [107]. COMMD1 interacts with the ECS–SOCS1 complex (composed of Elongins B/C, SOCS1, and Cullin2), promoting NF-κB ubiquitination and RelA degradation, thereby inhibiting NF-κB-mediated transcription [112] (Fig. 5). As a regulator of copper transport pathways, COMMD1 specifically interacts with NF-κB subunits to inhibit their transcriptional activity [113,114]. Notably, COMMD1 occupancy at promoter sites persists even after RelA removal, suggesting that COMMD1 inhibits NF-κB target gene expression through this mechanism [115]. Overexpression of COMMD1 reduces RelA chromosomal binding time and IκB ubiquitination, thereby suppressing IBD onset [116,117].

Reduced COMMD1 expression in circulating leukocytes of IBD patients leads to heightened NF-κB transcriptional activity, while COMMD1 deficiency in myeloid cells exacerbates inflammatory responses. Murine models reveal that myeloid-specific COMMD1 deficiency exacerbates LPS-induced IBD manifestations [118]. This highlights the anti-inflammatory role of COMMD1, with its deficiency potentially increasing the risk of colitis-associated cancer. Cu-related drugs and proteins, such as tetrathiomolybdate (TTM; a potential NF-κB transcription inhibitor), transgenic overexpression of Cu/ZnSOD (shown to alleviate DSS-induced colitis), and GPx1/GPx2 (or GSH biosynthesis inhibitors like buthionine sulfoximine), hold promise as therapeutic options for reducing inflammation and treating IBD [107,119,120].

#### Modulating the function of HIF-1α

Inflammation represents a fundamental pathological characteristic of IBD, and hypoxia is considered a normal phenomenon in most inflammatory processes. HIF-1α functions as a central regulator that facilitates intestinal cellular adaptation to hypoxia and preserves intestinal homeostasis [121]. HIF-1α exerts broad physiological effects, triggering transcription by binding to hypoxia response elements (HREs) in various genes [122]. This regulation of cellular hypoxic responses is essential for both physiological adaptation to low oxygen and inflammatory modulation [123,124]. Since HIF-1α enhances intestinal epithelial barrier function, it is a potential therapeutic target for addressing epithelial barrier dysfunction in IBD.

**Fig. 6.**
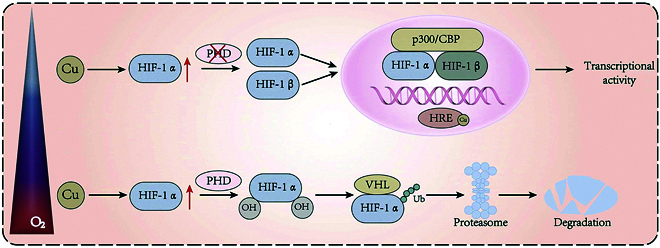
Regulation mechanism of HIF-1α stability and transcriptional activity under different oxygen concentrations and Cu ion levels. Under normoxic conditions, HIF-1α undergoes hydroxylation mediated by prolyl hydroxylase (PHD), which facilitates its recognition by the von Hippel–Lindau (VHL) protein. Then, the VHL protein subsequently ubiquitinates HIF-1α, marking it for proteasomal breakdown. However, in low-oxygen environments or with increased copper concentrations, HIF-1α hydroxylation is blocked, allowing its stabilization. The stabilized HIF-1α forms a dimer with HIF-1β and interacts with coactivators (like p300/CBP). This complex binds to hypoxia response elements (HREs) in the promoter regions of target genes, initiating the expression of adaptive genes. Additionally, Cu ions can further enhance HIF-1-mediated transcriptional activity by directly interacting with HREs, amplifying the hypoxia-induced gene expression response.

Research has demonstrated that Cu can enhance HIF-1α stability and facilitate its accumulation [125]. Moreover, copper potentiates HIF-1α accumulation and modulates its transcriptional activity by altering HRE binding in target genes [126–128]. The underlying mechanism suggests that copper, by facilitating the transport of the CCS (copper chaperone for superoxide dismutase 1 [SOD1]), ensures that HIF-1α can selectively bind to its target genes (e.g., BNIP3). Furthermore, the interaction dynamics between HIF-1α and the HREs within its gene targets is copper-dependent, given copper’s essential function in assembling the HIF-1 transcriptional machinery, and it amplifies the production of factors such as VEGF (vascular endothelial growth factor). In addition, elevated copper levels can stabilize HIF-1α, causing its cytoplasmic buildup and subsequent activation of the HIF-1 signaling cascade [129] (Fig. 6). Additionally, Cu participates in modulating the assembly of the HIF-1α transcriptional complex. In this context, Cu may inhibit HIF-1α activity to maintain the ability of HIF-1α to bind its cofactors. While Cu deprivation does not impact the expression of HIF-1α, excessive Cu can enhance the functionality of HIF-1α through mechanisms shared by other nonessential transition metals [126]. This enhancement enables cells to adapt to hypoxic and inflammatory environments, thereby influencing the onset, progression, and treatment of IBD.

## Bidirectional Regulation of Cu in CRC

**Fig. 7.**
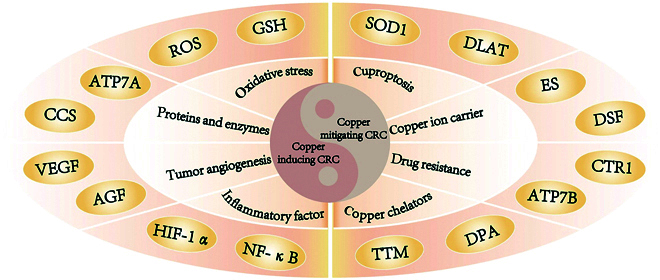
Schematic diagram of the promotion and inhibition effects of Cu metabolism and cuproptosis on CRC. This figure shows how various factors affect cancer processes including cell proliferation, apoptosis, angiogenesis, and immunity during CRC therapy.

Cu acts as a vital micronutrient, contributing significantly to core biological activities and systemic functions, particularly in enzymatic reactions, redox processes, and energy metabolism [130]. Cu homeostasis is crucial for maintaining proper cellular function [131]. Since the discovery of cuproptosis, researchers have reexamined the involvement of Cu and Cu-triggered cellular demise in tumorigenesis, bringing Cu back into focus in cancer research. CRC, a malignancy with high incidence and mortality rates, involves complex pathological mechanisms, with dysregulated copper metabolism and cuproptosis emerging as critical areas of investigation. Research has demonstrated that aberrant Cu accumulation can drive CRC progression by triggering oxidative stress, disrupting DNA repair mechanisms, and stimulating cellular proliferation and metastatic activity. At the same time, Cu-mediated cell death mechanisms, such as inducing mitochondrial damage and intrinsic apoptosis, may exert inhibitory effects on tumor growth [34]. Collectively, the biphasic regulation of Cu homeostasis in CRC underscores its therapeutic potential as a druggable target. Elucidating this mechanism holds great importance for understanding the molecular pathology of CRC and designing targeted therapeutic approaches (Fig. 7).

### Pro-tumor effects of Cu

#### Tumor angiogenesis

Angiogenesis refers to the creation of neovascularization from established vasculature, supplying oxygen and vital nutrients to rapidly dividing tumor cells [132,133]. This process comprises multiple coordinated steps, including endothelial cell proliferation and migration, vascular basement membrane remodeling, and de novo vascular network formation [132]. McAuslan was the first to suggest the angiogenic-promoting characteristics of copper, noting that copper salts could stimulate the movement of vascular lining cells, which is a vital initial phase in the process of angiogenesis. Subsequent studies demonstrated that topical copper application induces neovascularization in the rabbit corneal model. Furthermore, research has shown that copper substantially enhances the growth and activity of vascular endothelial cells [134].

When tumor development exceeds the supply of existing blood vessels, the tumor tissue enters a hypoxic state. Under these conditions, intracellular copper stabilizes HIF-1α, thereby promoting angiogenesis. Excess Cu increases the NF-κB’s transcriptional activity, boosting the production of pro-angiogenic factors, thereby promoting blood vessel formation [135,136]. Cu homeostasis is regulated by proteins such as ATP7A, a Cu-exporting protein that maintains intracellular Cu levels to safeguard cells against copper toxicity. ATP7A expression is posttranscriptionally regulated by copper levels and facilitates copper delivery to oncogenesis-associated cuproenzymes, including those driving cell proliferation, metastasis, and angiogenesis [137]. Additionally, SOD1, a modulator of vascular constriction, can boost VEGF production and potentiate FGF (fibroblast growth factor)-driven angiogenesis, facilitating the formation of tumor-associated vasculature [34]. Likewise, antioxidant 1 (ATOX1), a copper chaperone, has emerged as a pivotal regulator in the angiogenic process. Silencing ATOX1 inhibits stimulation of migration in smooth muscle cells of blood vessels through the action of PDGF, suggesting its crucial role in tumor angiogenesis [138]. In summary, Cu’s ability to modulate multiple angiogenic factors highlights its potential to promote tumor progression by supporting new blood vessel formation essential for tumor growth.

#### Cu-dependent proteins driving cancer metastasis

Cu constitutes a fundamental element in numerous enzymes and proteins that are intricately involved in cancer biology, such as SOD1, lysyl oxidase (LOX), and CCS [139]. These Cu-dependent proteins promote cancer metastasis through activation of oncogenic enzymatic pathways. The copper chaperone CCS mediates the delivery of Cu ions to SOD1. SOD1 is essential for preserving intracellular redox balance. Upon binding zinc and copper ions, this enzyme demonstrates robust catalytic activity that facilitates superoxide anion radical dismutation, thereby reducing intracellular oxidative damage. Research shows that the regulation of Cu chaperones ATOX1 and CCS is elevated in CRC, uterine cancer, and liver cancer, leading to increased activity of Cu-dependent enzymes and promoting tumor cell proliferation and metastasis [140]. Additionally, LOX, an extracellular copper-dependent enzyme, is essential for mediating the covalent crosslinking of ECM components. LOX is acknowledged as a pro-metastatic factor in CRC, prostate cancer, and liver cancer, promoting cancer cell invasion and dissemination [141].

### Antitumor effects of Cu

#### Targeting tumor cells via cuproptosis

**Fig. 8.**
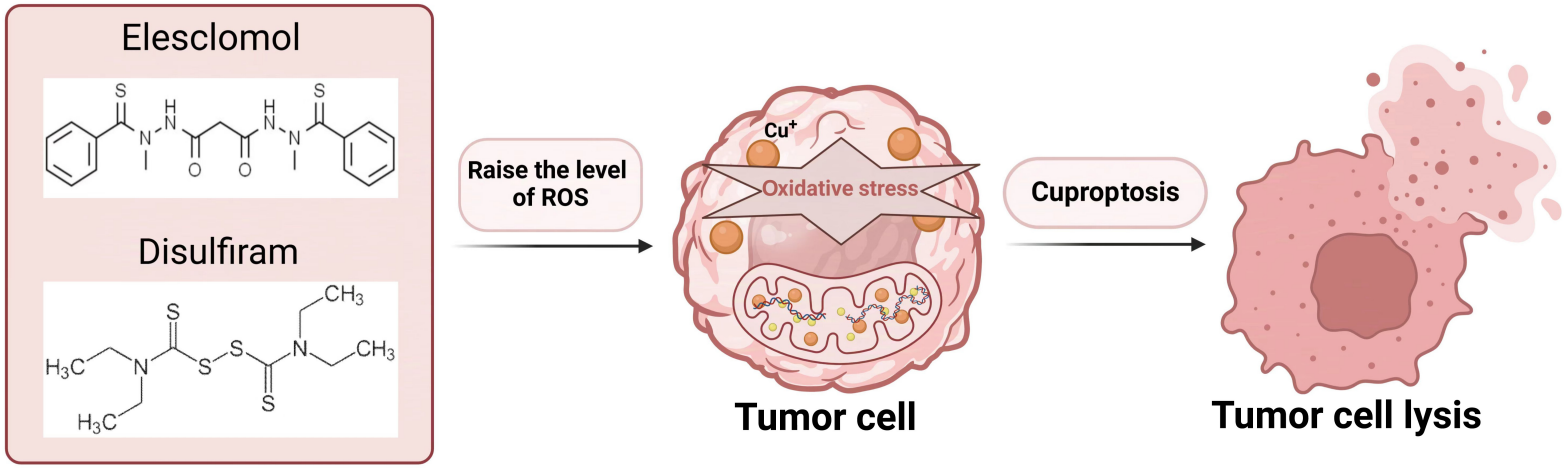
The figure illustrates the mechanism by which elesclomol and disulfiram induce cuproptosis in tumor cells via oxidative stress and Cu ion (Cu^+^) accumulation. ES and DSF elevate intracellular ROS concentrations, exacerbating oxidative stress, disrupting Cu ion homeostasis, and causing mitochondrial dysfunction. This ultimately triggers cuproptosis and promotes tumor cell death. This mechanism provides critical evidence for antitumor therapies targeting Cu ion metabolism.

Disrupting the balance of ROS in cells triggers oxidative stress responses [142]. Evidence indicates that oncogenic signaling and mutations increase ROS production, which is closely associated with cancer initiation and progression. Growth factor signaling promotes ROS generation, thereby accelerating tumor progression [143–146]. ES and DSF, as copper ion carriers, selectively transport Cu to mitochondria, leading to localized ROS accumulation and inducing tumor cell death [147] (Fig. 8). The specific mechanism involves mitochondrial FDX1 reducing Cu^2+^ (carried by ES) to Cu^+^. The liberated Cu^+^ reacts with oxygen molecules, generating superoxide radicals, which subsequently dismutate into hydrogen peroxide. Hydrogen peroxide further reacts with Cu^+^ to generate highly destructive hydroxyl radicals. This cascade of reactions significantly elevates ROS levels, ultimately inducing tumor cell death [148]. Furthermore, Cu ions complexed with molecular carriers catalyze hydroxyl radical formation, amplifying oxidative stress and inducing tumor cell apoptosis. Additionally, Cu-binding proteins such as ceruloplasmin can be directed toward increase oxidative stress levels and induce tumor cell death [149]. Cuproptosis offers a tumor-suppressive mechanism, providing new opportunities for CRC treatment. It is anticipated that in the coming years, advancements in clinical diagnostics and therapeutics will further harness cuproptosis for cancer therapy.

#### Cu chelators regulating cancer cell immune evasion

Cancer immune evasion is widely acknowledged as one of the core characteristics of tumor progression, where tumor cells overexpress PD-L1 (programmed death-ligand 1) to escape immune surveillance. PD-L1, an immune checkpoint protein expressed on tumor cells, mediates immune evasion by suppressing antitumor immune responses [34]. Research has shown that Cu enhances PD-L1 expression in tumor cells, thus enhancing their immune escape capacity. Intracellular Cu up-regulates PD-L1 transcription and stabilizes its protein levels. Compared to normal tissues, elevated Cu levels in cancer cells significantly enhance PD-L1 expression [150].

Conversely, Cu-chelating agents reduce PD-L1 levels by diminishing tumor cell sensitivity to inflammatory signaling molecules like interferon γ (IFNγ), TNF-α, and IFNα/β. Cu deprivation down-regulates the activation through phosphorylation of STAT3 (signal transducer and activator of transcription 3), EGFR (epidermal growth factor receptor), AKT (AKT serine/threonine kinase), and GSK3β (glycogen synthase kinase 3 beta), thereby inhibiting PD-L1 transcription and modulating PD-L1 ubiquitination and stability. Cu ion carriers like DSF can increase PD-L1 stability through Cu overload [151], while Cu chelators demonstrate the potential to enhance antitumor immune responses. For instance, TTM suppresses MEK1/2 kinase activity via copper chelation, thereby inhibiting CRC progression [152]. The tumor-suppressing activity of Cu chelators has been confirmed in various cancers, like CRC and breast cancer [34]. By targeting Cu-dependent pathways involved in immune evasion, Cu chelators could provide a novel therapeutic strategy to boost antitumor immunity.

## Cu metabolism and cuproptosis in the clinical application of IBD and CRC

### Inflammatory bowel disease

Currently, most IBD cases are incurable, with treatment focused on disease control and relapse prevention, often requiring surgery. Current therapeutic approaches predominantly utilize pharmacological agents including antibiotics, aminosalicylates, corticosteroids, and immunomodulators [153]. Recent progress in IBD research have highlighted the function of Cu metabolism and cuproptosis in IBD pathogenesis, offering novel therapeutic approaches.

#### Antioxidants

Research suggests that elevated ROS levels and reduced antioxidant defenses contribute to IBD development. Antioxidants play a critical role in IBD pathophysiology and are categorized into catalytic and noncatalytic groups based on their mechanisms of action. In the enzymatic antioxidant defense system, ATOX1 serves as a Cu chaperone and has been shown to be indispensable for maintaining intracellular Cu homeostasis, counteracting oxidative stress, and regulating gene transcription [154]. These properties establish ATOX1 as a promising therapeutic target for mitigating intestinal inflammation through dual modulation of copper homeostasis and antioxidant pathways in IBD management. Within the nonenzymatic antioxidant defense mechanisms, GSH, the most prevalent thiol in the cytoplasm, serves an essential role. GSH protects cells by scavenging free radicals and inhibiting pro-oxidants. It additionally acts as a critical component for various enzymatic reactions involved in antioxidant defense and detoxification processes, such as GPx, glutathione S-transferase (GST), and glyoxalase [83]. Studies suggest using antioxidants as an alternative therapy to anti-inflammatory drugs or immunomodulators for patients with uncomplicated gastrointestinal diseases [95]. This approach could provide more effective treatment options for IBD patients, reducing the side effects of long-term anti-inflammatory drug use and facilitating personalized treatment strategies.

#### Regulation of Cu proteins and pathway activities

COMMD1 is involved in various cellular mechanisms, including Cu transport, ion balance, and hypoxic responses [155]. In IBD, NF-κB dissociates from its inhibitory complex with IκB and translocates to the nucleus, where it up-regulates proinflammatory gene expression. Normally, newly synthesized IκB promotes NF-κB nuclear export to terminate the activation process. Unlike IκB, COMMD1 works in conjunction with the ubiquitin ligase Cul2, targeting nuclear-localized NF-κB for ubiquitination and subsequent proteasomal degradation, thus acting as an inhibitor of NF-κB [156]. Research indicates that inflammation suppresses COMMD1 production, creating a reinforcing feedback loop that persistently activates NF-κB, exacerbating inflammation [156]. In IBD patients, COMMD1 expression is reduced in inflamed tissues and circulating leukocytes. Colitis mouse models exhibit reduced COMMD1 expression, indicating an inflammation-mediated response that may sustain chronic inflammation [157]. Clinically, restoring or enhancing COMMD1 expression could significantly impact IBD treatment. Strategies such as inhibiting the NF-κB pathway, blocking persistent inflammatory signaling, or developing drugs targeting COMMD1 expression or function could help suppress inflammation.

#### Diagnosing IBD using Cu levels

IBD is prone to misdiagnosis, particularly in individuals with ileal Crohn’s disease or those under 40 years of age [158]. Cu levels in the body can aid in diagnosis. Studies have found a significant correlation between the Cu-to-zinc ratio and biomarkers like C-reactive protein and calprotectin in active IBD patients. Patients with Crohn’s disease demonstrate significantly higher serum copper concentrations (22.7 ± 5.49 μmol/l) compared to both ulcerative colitis patients (17.6 ± 5.15 μmol/l) and healthy controls (20.76 ± 4.06 μmol/l; *P* < 0.01) [159]. These results indicate that Cu levels may function as a preliminary diagnostic marker for IBD.

### Colorectal cancer

Recent studies have identified a strong connection between Cu and cancer, with abnormal Cu levels emerging as a new therapeutic target. Cu concentrations in the blood of individuals with cancer are significantly higher than in healthy individuals, often exceeding 10 times the normal levels. Thus, intracellular Cu levels are closely related to tumorigenesis and progression [160].

#### Cu-targeted therapy for CRC

COX17 is a critical Cu chaperone in the IMS. It facilitates Cu transfer from the cytoplasm to the mitochondrial IMS, aiding in the formation of COX [161,162]. Specifically, COX17 delivers Cu to mitochondrial membrane proteins SCO1 and SCO2, facilitating its incorporation into COX2 [163]. COX17 also transports Cu to COX11 for insertion into COX1 [163,164]. Targeting COX17 emerges as a viable therapeutic strategy for eradicating both solid and hematologic malignancies [165]. In extracellular Cu transport, ATP7A and ATP7B are key Cu-exporting proteins. Under conditions of copper deficiency, ATP7A/B resides within the trans-Golgi network (TGN), enabling the movement of copper from the cellular fluid into the TGN. As Cu levels increase, ATP7A and ATP7B serve as essential proteins for copper exportation. In conditions of copper scarcity, they are localized within the TGN, aiding in the movement of Cu from the cytosol to the lumen of the TGN. After copper levels return to normal concentrations, ATP7A/B are recycled and return to the TGN. Simultaneously, under conditions of increased copper concentration, ATP7A/B undergoes from the TGN to either the cellular periphery or vesicular compartments that subsequently merge with the plasma membrane, facilitating copper efflux. Following restoration of homeostatic copper levels, ATP7A/B undergo retrograde trafficking to the TGN. Additionally, these proteins assist in transporting copper ions from the TGN to copper-laden post-Golgi vesicles. These vesicular compartments ultimately fuse with the plasma membrane through membrane fusion events, facilitating the export of copper ions to the extracellular space. This mechanism of copper excretion is evident across various cell types, including cancerous ones [34]. In hepatic cells, ATP7B specifically directs copper from secretory vesicles into bile, thereby regulating copper levels and preventing excessive accumulation. In CRC cells harboring KRAS (Kirsten rat sarcoma viral oncogene homolog) mutations, ATP7A up-regulation inhibits Cu-induced toxicity caused by excess Cu [166]. This implies that targeting ATP7A might act as a synthetic lethal strategy to eliminate KRAS-mutant CRC cells.

#### Cuproptosis combined with other therapies

Targeting cuproptosis can also be combined with chemotherapy, radiotherapy, nanotechnology, photothermal therapy (PTT), and chemodynamic therapy to enhance antitumor effects [167,168]. Recent studies have established the feasibility of engineering nanodrug delivery systems for tumor-specific Cu or Cu ionophore release, inducing localized cuproptosis and enhancing treatment efficacy [169]. For example, E-C@DOX NPs contain Cu^2+^ and the frontline chemotherapeutic agent doxorubicin (DOX). This combination induces cuproptosis while suppressing signaling pathways linked to tumor stemness and survival, enhancing the therapeutic effect [170]. Similarly, LDH (lactate dehydrogenase)/HA (hyaluronic acid)/5-fluorouracil (5-FU) nanosheets are designed to act on malignant cells releasing Cu^2+^ and 5-FU, thereby effectively inducing cuproptosis and demonstrating strong tumor suppression effects [171]. Another nanoplatform, ZnPc*/Cu/SN38@NP, slowly breaks down via protonation of carboxylic acid groups or binding with Cu^2+^ by GSH. This process disrupts the relationships among the Cu^2+^-induced NPs, ultimately releasing unbound ZnPc* and SN38. Studies have shown that ZnPc* exerts a photodynamic therapy (PDT) effect, while SN38 provides chemotherapeutic activity, resulting in a synergistic dual antitumor effect [172]. Furthermore, a novel GSH-responsive nanodrug, Es@CuTCPP, was developed by loading ES onto CuTCPP nanosheets. After accumulation at the tumor site, Es@CuTCPP not only becomes highly sonosensitive upon reaction with the overexpressed GSH in the TME but also forms CuEs complexes through the co-released Cu^2+^ ions and chelated Es. Consequently, Es@CuTCPP enables the generation of ROS in cancer cells upon ultrasound irradiation and modulates copper metabolism, triggering substantial cuproptosis. In a CT26 colon cancer subcutaneous xenograft model, systemic administration experiments confirmed the excellent tumor-targeting capability of Es@CuTCPP, with no significant systemic toxicity or major organ damage observed. These findings robustly demonstrate that Es@CuTCPP exhibits favorable biosafety and promising potential for clinical translation [173].

PTT is a noninvasive oncological therapy in which photothermal agents absorb ambient light energy—particularly near-infrared (NIR) radiation—and transform it into heat to target and destroy tumor cells [174]. Research indicates that LPS-CuS NPs hold significant potential in immunophotothermal therapy, effectively eradicating primary CRC and completely preventing metastasis to the spleen and liver. Photothermally responsive nanoplatforms (e.g., Au@MSN-Cu/PEG/DSF) exhibit synergistic antitumor effects when combined with PTT, enabling efficient cancer cell eradication and tumor growth suppression [167]. These findings underscore the strong connection between Cu metabolism and CRC development, treatment, and prognosis. Combining cuproptosis with other therapeutic strategies to modulate Cu ion levels represents a promising approach for CRC treatment. This synergistic strategy not only enhances antitumor effects but also provides a potential avenue for improving patient outcomes.

#### Overcoming drug resistance with Cu-based drugs

Despite the advancement of numerous novel approaches to cancer therapy over the past few years, chemotherapy continues to be the cornerstone of cancer therapy. Platinum-based agents constitute one of the most widely employed classes of chemotherapeutics in oncology. Nevertheless, a significant obstacle in chemotherapy is the onset of therapeutic resistance, often diminishing its therapeutic impact [175,176]. Emerging evidence suggests that combining chemotherapeutic drugs with Cu ion carriers can overcome resistance. In copper ionophores and copper-based drugs, DSF and ES have received the most attention and have undergone clinical trials. In most current clinical trials involving ES and DSF, no clinical benefits were observed in unselected populations, but their safety profiles were comprehensively evaluated (Table 4).

**Table 4.** Clinical safety assessment of copper-based therapeutic agents

NCT number	Phases	Participant number	Status	Conditions	Drugs	Result
NCT05210374	Phase 1	24	Recruiting	Relapsed sarcomas	Disulfiram, copper gluconate, liposomal doxorubicin	–
NCT03363659	Phase 2	15	Terminated	Glioblastoma, glioblastoma multiforme	Disulfiram, copper gluconate, temozolomide	Only one case of adverse events was observed.
NCT03034135	Phase 2	23	Completed	Recurrent glioblastoma	Disulfiram/copper, temozolomide	–
NCT03714555	Phase 2	1	Completed	Metastatic pancreatic cancer	Disulfiram, copper gluconate	One adverse event was reported in the Nab-Paclitaxel/Gemcitabine plus DSF/Cu treatment group.
NCT03323346	Phase 2	150	Recruiting	Breast neoplasm female, metastatic breast cancer	Disulfiram	–
NCT00742911	Phase 1	21	Completed	Cancer	Disulfiram, copper gluconate	The regimen demonstrated good tolerability with no observed objective responses.
NCT02770378	Phase 1 Phase 2	10	Completed	Glioblastoma	Aprepitant, auranofin, captopril, celecoxib, disulfiram, itraconazole, minocycline, ritonavir, sertraline	Nine drug combinations including DSF were safely administered under monitoring
NCT00522834	Phase 3	630	Terminated	Melanoma	Elesclomol (STA-4783), paclitaxel	–
NCT00827203	Phase 1	30	Suspended	Metastatic solid tumors	Elesclomol sodium	With a favorable safety profile
NCT00888615	Phase 2	58	Completed	Fallopian tube clear cell adenocarcinoma, fallopian tube endometrioid adenocarcinoma, fallopian tube mucinous adenocarcinoma, fallopian tube serous adenocarcinoma	Elesclomol sodium, paclitaxel	With a favorable safety profile
NCT00808418	Phase 1	34	Completed	Prostate cancer	Elesclomol sodium, docetaxel	–
NCT00084214	Phase 1	103	Completed	Melanoma	Elesclomol (STA-4783), paclitaxel	Median progression-free survival doubled with manageable toxicity.
	Phase 2					
NCT00087997	Phase 2	80	Completed	Soft tissue sarcoma	Elesclomol (STA-4783)	–
NCT00088114	Phase 1	50	Completed	Neoplasms	Elesclomol (STA-4783), paclitaxel	–
NCT00088088	Phase 1	86	Completed	Stage IIIB non-small cell lung cancer, stage IV non-small cell lung cancer	Paclitaxel, carboplatin, elesclomol (STA-4783)	–
	Phase 2					

For example, the combination of 5-FU and DSF/Cu has been demonstrated to effectively eliminate tumor cells in CRC. DSF/Cu down-regulates indicators linked to 5-FU resistance, including thymidylate synthase and CD133/CD44 [177]. Similarly, the ES–Cu complex exerts potent anticancer effects through copper-dependent mechanisms, including ROS accumulation and subsequent tumor cell death, demonstrating multimodal cytotoxic activity against malignant cells [16,178,179]. Both approaches have demonstrated significant antitumor effects in preclinical experiments; however, clinical trials have yet to yield satisfactory results [180]. One potential restrictive factor could be the challenge of maintaining persistently elevated Cu concentrations within cancer cells in clinical settings. Conducting further studies could facilitate the transition of Cu-based drugs from experimental research to clinical application, thereby providing a more effective means of overcoming tumor resistance (Table 5).

**Table 5.** Cu-based anti-CRC agents

Compound	Cancer type	Materials	Antitumor effects and their underlying mechanisms	Reference
DDTC	CRC	HCT116	Suppression of PKM2-driven aerobic glycolysis	[215]
Elesclomol/Cu	CRC	SW480, DLD-1	Elesclomol triggers copper-mediated ferroptosis in colorectal cancer cells by promoting the breakdown of ATP7A and SLC7A11	[216]
Disulfiram	CRC	H630WT, DLD-1	Disulfiram suppresses both constitutive and 5-fluorouracil (5-FU)-activated NF-κB pathways. It amplifies the cell-killing effects of 5-FU in DLD-1 and RKOWT cells and successfully overcomes 5-FU resistance in the H630 5-FU-resistant cell line	[217]
Disulfiram/Cu	CRC	RKO, Ht29	Disulfiram–copper significantly decreases colorectal cancer cell survival, predominantly triggering autophagy over apoptosis through the up-regulation of ULK1	[218]
Disulfiram/Cu	CRC	HCT116, SW620, HCT8	Disulfiram–copper can suppress colorectal cancer cell proliferation, possibly via the activation of ICD	[219]
Disulfiram/Cu	CRC	HCT116	Disulfiram through its metabolite CuDDC significantly lowers cellular H₂S concentrations and significantly inhibits the growth of HCT116 cells	[220]
Curcumin	CRC	Caco-2	Curcumin modulates lipid, RNA, NADH, and NADPH metabolism, increases the expression of positive cuproptosis mediators, and promotes cuproptosis in CRC cells	[221,222]
4-OI	CRC	HCT116, LoVo	4-OI targets GAPDH to inhibit glycolysis, thereby potentiating elesclomol–Cu-induced cuproptosis	[181]
TPEN	CRC	Caco-2	The cytotoxicity induced by TPEN was alleviated by the supplementation of Cu^2+^. Down-regulation of ATOX1 increased the susceptibility of Caco-2 cells to TPEN-induced toxicity	[223]
TPEN	CRC	SW480, HT-29, LOVO	TPEN chelates Cu to form TPEN–Cu complexes, which engage in redox cycling, leading to selective targeting and elimination of CRC cells	[224]
JYFY-001	CRC	SW620, HCT116, HLF-1	In vitro: Reduces proliferation of cancer cells, induces apoptosis, and decreases both the rate of extracellular acidification and oxygen utilization in CRC cells. In vivo: Inhibits the growth of CRC xenograft tumors, increases tumor cell apoptosis, enhances lymphocyte infiltration, and potentiates the antitumor efficacy of PD-1 inhibitors	[225]
Melon	CRC	HTB-35, HT-29	Melon exhibits growth-inhibiting properties in multiple human cancer cell lines, including renal, colorectal, and cervical cancers, through its copper-chelating function	[226]
COTI-NH2	CRC	SW480	Buthionine sulfoximine treatment significantly sensitized cells to COTI-NH2, showing a comparable effect in both SW480 and SW480/Coti cells	[227]
DTN	CRC	HCT116	Through the mechanism of ROS-dependent ubiquitination that facilitates Mcl-1 degradation, the combinatorial treatment with 5-FU, DTN, and DHA exhibited potent pro-apoptotic effects in colorectal cancer cells, ultimately manifesting as marked tumor volume reduction and weight loss	[228]
HydroCuP	CRC	CT-15, LoVo	HydroCuP demonstrated remarkable efficacy in suppressing tumor progression with negligible toxic effects in preclinical models. Notably, in human colorectal cancer xenografts, HydroCuP exhibited potent therapeutic activity across both oxaliplatin-sensitive and oxaliplatin-resistant tumor variants	[229]

#### Small-molecule compounds inducing cuproptosis

Several small-molecule compounds have been identified that may increase tumor cell sensitivity to cuproptosis. Emerging studies have identified multiple bioactive compounds capable of triggering cuproptosis, a novel form of regulated cell death mediated by copper toxicity. For example, in CRC cells, 4-OI induces Cys22 alkylation of GAPDH, targeting GAPDH to suppress its activity. This inhibition disrupts glycolysis in tumor cells, promoting electrochlorination-mediated Cu precipitation. In vivo studies demonstrate that ES containing 4-OI significantly suppresses tumor growth, exhibiting potent antitumor activity. In addition, 4-OI continues to promote cuproptosis even following Nrf2 knockdown, validating its antitumor effects through GAPDH targeting [181].

## Conclusion and Outlook

Cell death is essential for preserving physiological equilibrium and maintaining tissue integrity; however, it may also represent a maladaptive reaction to harmful triggers [182]. To date, more than 10 distinct modes of cell death have been identified, including apoptosis, pyroptosis, and ferroptosis [183]. Since the identification of cuproptosis in 2022, this recently discovered cellular death pathway has garnered significant research interest due to its dual role in IBD and CRC [16]. Cu, as a vital trace element, exhibits dual roles in metabolism and is particularly critical for maintaining intestinal health [184]. On one hand, appropriate copper levels are considered to effectively suppress inflammatory responses associated with IBD. Copper can modulate inflammatory pathways such as NF-κB to inhibit excessive immune reactions and selectively eliminate transformed cells through cuproptosis. The maintenance of this homeostasis is critical for intestinal epithelial barrier function and stem cell niche balance. This protective mechanism may be related to copper’s regulation of inflammatory signaling pathways or its induction of cuproptosis in tumor cells, thereby partially suppressing tumor initiation and progression. On the other hand, dysregulated copper metabolism or abnormally elevated copper levels may exacerbate IBD pathology and create favorable conditions for CRC development by promoting oxidative stress, stimulating angiogenesis, and compromising intestinal epithelial barrier integrity. This mechanism is closely linked to gut microbiota dysbiosis, as impaired barrier function further disrupts intestinal homeostasis maintained by commensal bacteria and the immune system. Studies by Roy et al. [185] have shown that IBD-associated gut microbial communities may promote disease progression through immune cell-mediated intestinal barrier damage. Thus, copper dysregulation may disrupt this delicate balance, aggravating microbial dysbiosis and immune dysfunction, ultimately forming a pathological cycle that drives IBD progression. Notably, this bidirectional regulation exhibits significant tissue specificity, with intestinal epithelial cells displaying a higher tolerance threshold for copper. Moreover, long-term low-dose exposure may induce cumulative effects resembling acute toxicity. These findings not only highlight the pivotal role of copper homeostasis in CRC pathogenesis but also provide a molecular basis for developing precision therapeutic strategies targeting copper modulation. This underscores the need for establishing individualized copper monitoring systems in clinical interventions. These findings underscore the importance of Cu metabolism homeostasis in maintaining intestinal immune equilibrium and preventing related diseases, warranting further investigation into its underlying mechanisms. Consequently, modulating copper ion homeostasis has emerged as a promising therapeutic approach for both IBD and CRC, demonstrating substantial translational potential. Compared to conventional types of cell death, cuproptosis has garnered interest due to its ability to reprogram the immune microenvironment. Cuproptosis may reshape the immunologic characteristics of the TME, thereby modulating its capacity to inhibit tumor progression and metastatic dissemination [186]. Studies suggest that tumor immunity within the TME can be modulated by cuproptosis [35]. For example, genomic analysis of 1,274 CRC specimens uncovered copper-associated molecular signatures comprising 16 core cuproptosis regulators, establishing a connection between TME heterogeneity and copper dysregulation patterns [187]. Cu-complexed NPs, such as Cu(I)NPs, have been shown to induce cuproptosis, triggering ICD, activating adaptive immune responses, facilitating DC maturation, and boosting CD8^+^ T-cell infiltration into tumor tissues. This process enhances antitumor immunity and remodels the immune microenvironment [58]. While it is speculated that cuproptosis contributes to shape antitumor immunity, whether Cu-dependent cell death suppresses cancer immunotherapy remains uncertain. Consequently, understanding the pathways of cuproptosis is crucial for progressing innovative combination therapies. Traditional targeted therapies induce the death of malignant cells by disrupting specific cancer-related pathways or molecular targets. Recent medical advances have led to the development of innovative therapeutic strategies and pharmacological agents. For example, the combination of 5-FU and DSF/Cu effectively eliminates CRC cells, LPS-CuS achieves primary CRC eradication through immunophotothermal therapy, and Cu NPs promote tumor antigen presentation and stimulate specific immune responses [34]. Therefore, the dual role of Cu metabolism and cuproptosis presents broad prospects for personalized immunotherapy in IBD and CRC.

As a newly identified mechanism of cellular demise, cuproptosis is undergoing rigorous investigation across diverse disciplines, including chemotherapy, TME regulation, immune-based therapies, and outcome prediction, to develop more effective cancer management strategies. However, the study of cuproptosis is still a nascent field, with current studies primarily focused on its correlation with IBD and CRC. Numerous underlying mechanisms remain to be elucidated, necessitating additional fundamental investigations. Future investigations should focus on deciphering the precise molecular pathways that govern Cu metabolism and cuproptosis in the context of IBD and CRC, as well as explore strategies to modulate Cu levels to maximize therapeutic efficacy. Despite its potential, several challenges remain in cuproptosis research, which also pose obstacles for clinical applications. First, the potential clinical applications and safety concerns related to cuproptosis modulation remain to be fully explored, posing significant challenges for the clinical translation of cuproptosis inducers. Both copper deficiency and excess can induce systemic toxicity, necessitating additional clinical studies to evaluate how copper level modulation affects prognosis in IBD and CRC patients. Such investigations are crucial for improving the targeting efficiency and in vivo stability of these therapeutic agents. Second, Cu’s potential as a therapeutic target needs further exploration. This includes developing therapies based on Cu metabolism and cuproptosis, such as gene knockouts and cell-based approaches, while research efforts are shifting toward novel copper inducers, including plant-derived compounds, synthetic molecules, and nanotechnology-based carriers, to improve targeted drug delivery to affected cells. Additionally, the connection between copper metabolism, cuproptosis, and the intestinal immune microenvironment requires further investigation. Combining cuproptosis with immunotherapy could offer a promising strategy to combat IBD and CRC, significantly improving treatment outcomes and extending patient survival. However, it remains unclear whether cuproptosis and its signaling pathways play a protumor role in tumor initiation and development. The current lack of validated cuproptosis biomarkers underscores the need for additional research to enable precision therapeutic interventions. Comprehending the interplay between cuproptosis and alternative forms of cellular demise constitutes another pivotal research domain. Investigating the connections between cuproptosis and other kind of pathways, including apoptosis, ferroptosis, and pyroptosis, may deepen our comprehension of Cu-related diseases and accelerate the progression of targeted therapies to induce tumor cell death more effectively. Looking forward, the discovery of specific biomarkers and personalized antitumor strategies will likely enable the clinical translation of therapies based on Cu metabolism and cuproptosis.

In summary, cuproptosis represents a potential novel therapeutic avenue for IBD and CRC. Advancing understanding of cuproptosis regulation and enhancing its induction efficiency position this pathway as a novel therapeutic strategy for disease intervention. This strategy has the potential not only to suppress disease onset and progression effectively but also to improve patient survival and quality of life through precision therapies. Therefore, understanding the involvement of cuproptosis in pathological processes and developing related treatment strategies holds substantial scientific and medical importance.

## References

[1] Xavier RJ, Podolsky DK. Unravelling the pathogenesis of inflammatory bowel disease. Nature. 2007;448:427–434. 17653185 10.1038/nature06005

[2] Bray F, Ferlay J, Soerjomataram I, Siegel RL, Torre LA, Jemal A. Global cancer statistics 2018: GLOBOCAN estimates of incidence and mortality worldwide for 36 cancers in 185 countries. CA Cancer J Clin. 2018;68(6):394–424. 30207593 10.3322/caac.21492

[3] Swann JB, Smyth MJ. Immune surveillance of tumors. J Clin Invest. 2007;117(5):1137–1146. 17476343 10.1172/JCI31405 PMC1857231

[4] Geremia A, Biancheri P, Allan P, Corazza GR, Di Sabatino A. Innate and adaptive immunity in inflammatory bowel disease. Autoimmun Rev. 2014;13(1):3–10. 23774107 10.1016/j.autrev.2013.06.004

[5] Jin K, Ren C, Liu Y, Lan H, Wang Z. An update on colorectal cancer microenvironment, epigenetic and immunotherapy. Int Immunopharmacol. 2020;89(Part A): Article 107041. 33045561 10.1016/j.intimp.2020.107041

[6] Roda G, Chien Ng S, Kotze PG, Argollo M, Panaccione R, Spinelli A, Kaser A, Peyrin-Biroulet L, Danese S. Crohn’s disease. Nat Rev Dis Primers. 2020;6(1):22. 32242028 10.1038/s41572-020-0156-2

[7] Kobayashi T, Siegmund B, le Berre C, Wei SC, Ferrante M, Shen B, Bernstein CN, Danese S, Peyrin-Biroulet L, Hibi T. Ulcerative colitis. Nat Rev Dis Primers. 2020;6(1):74. 32913180 10.1038/s41572-020-0205-x

[8] Wu H, Shen B. Pouchitis: Lessons for inflammatory bowel disease. Curr Opin Gastroenterol. 2009;25(4):314–322. 19349860 10.1097/MOG.0b013e32832b36eb

[9] Cleynen I, Boucher G, Jostins L, Schumm LP, Zeissig S, Ahmad T, Andersen V, Andrews JM, Annese V, Brand S, et al. Inherited determinants of Crohn’s disease and ulcerative colitis phenotypes: A genetic association study. Lancet. 2016;387(10014):156–167. 26490195 10.1016/S0140-6736(15)00465-1 PMC4714968

[10] Ionescu VA, Gheorghe G, Bacalbasa N, Chiotoroiu AL, Diaconu C. Colorectal cancer: From risk factors to oncogenesis. Medicina (Kaunas). 2023;59(9):1646. 37763765 10.3390/medicina59091646 PMC10537191

[11] Dekker E, Tanis PJ, Vleugels JLA, Kasi PM, Wallace MB. Colorectal cancer. Lancet. 2019;394:1467–1480. 31631858 10.1016/S0140-6736(19)32319-0

[12] Rubin DC, Shaker A, Levin MS. Chronic intestinal inflammation: Inflammatory bowel disease and colitis-associated colon cancer. Front Immunol. 2012;3:107. 22586430 10.3389/fimmu.2012.00107 PMC3347037

[13] Xu L, He B, Sun Y, Li J, Shen P, Hu L, Liu G, Wang J, Duan L, Zhan S, et al. Incidence of inflammatory bowel disease in urban China: A nationwide population-based study. Clin Gastroenterol Hepatol. 2023;21(13):3379–3386.e3329. 37660767 10.1016/j.cgh.2023.08.013

[14] Wungjiranirun M, Sharzehi K. Wilson’s disease. Semin Neurol. 2023;43:626–633. 37607588 10.1055/s-0043-1771465

[15] Türner Z, Horn N. Menkes disease: Recent advances and new insights into copper metabolism. Ann Med. 1996;28(2):121–129. 8732640 10.3109/07853899609092936

[16] Tsvetkov P, Coy S, Petrova B, Dreishpoon M, Verma A, Abdusamad M, Rossen J, Joesch-Cohen L, Humeidi R, Spangler RD, et al. Copper induces cell death by targeting lipoylated TCA cycle proteins. Science. 2022;375(6586):1254–1261. 35298263 10.1126/science.abf0529 PMC9273333

[17] Xie J, Yang Y, Gao Y, He J. Cuproptosis: Mechanisms and links with cancers. Mol Cancer. 2023;22(1):46. 36882769 10.1186/s12943-023-01732-y PMC9990368

[18] da Silva DA, de Luca A, Squitti R, Rongioletti M, Rossi L, Machado CML, Cerchiaro G. Copper in tumors and the use of copper-based compounds in cancer treatment. J Inorg Biochem. 2022;226: Article 111634. 34740035 10.1016/j.jinorgbio.2021.111634

[19] Ge EJ, Bush AI, Casini A, Cobine PA, Cross JR, DeNicola GM, Dou QP, Franz KJ, Gohil VM, Gupta S, et al. Connecting copper and cancer: From transition metal signalling to metalloplasia. Nat Rev Cancer. 2022;22:102–113. 34764459 10.1038/s41568-021-00417-2 PMC8810673

[20] Li Y. Copper homeostasis: Emerging target for cancer treatment. IUBMB Life. 2020;72(9):1900–1908. 32599675 10.1002/iub.2341

[21] Wadhwa S, Mumper RJ. D-penicillamine and other low molecular weight thiols: Review of anticancer effects and related mechanisms. Cancer Lett. 2013;337:8–21. 23727371 10.1016/j.canlet.2013.05.027

[22] Lin Q, Hou S, Dai Y, Jiang N, Lin Y. Monascin exhibits neuroprotective effects in rotenone model of Parkinson’s disease via antioxidation and anti-neuroinflammation. Neuroreport. 2020;31(9):637–643. 32427711 10.1097/WNR.0000000000001467

[23] Zhang S, Zong Y, Chen L, Li Q, Li Z, Meng R. The immunomodulatory function and antitumor effect of disulfiram: Paving the way for novel cancer therapeutics. Discov Oncol. 2023;14:103. 37326784 10.1007/s12672-023-00729-9 PMC10275851

[24] Festa RA, Thiele DJ. Copper: An essential metal in biology. Curr Biol. 2011;21(21):R877–R883. 22075424 10.1016/j.cub.2011.09.040 PMC3718004

[25] Bost M, Houdart S, Oberli M, Kalonji E, Huneau JF, Margaritis I. Dietary copper and human health: Current evidence and unresolved issue s. J Trace Elem Med Biol. 2016;35:107–115. 27049134 10.1016/j.jtemb.2016.02.006

[26] Lutsenko S. Dynamic and cell-specific transport networks for intracellular copper ions. J Cell Sci. 2021;134(21):jcs240523. 34734631 10.1242/jcs.240523 PMC8627558

[27] Lönnerdal B. Intestinal regulation of copper homeostasis: A developmental perspective. Am J Clin Nutr. 2008;88(3):846S–850S. 18779306 10.1093/ajcn/88.3.846S

[28] Doguer C, Ha J, Collins J. Intersection of iron and copper metabolism in the mammalian intestine and liver. Compr Physiol. 2018;8(4):1433–1461. 30215866 10.1002/cphy.c170045 PMC6460475

[29] Migocka M. Copper-transporting ATPases: The evolutionarily conserved machineries for balancing copper in living systems. IUBMB Life. 2015;67(10):737–745. 26422816 10.1002/iub.1437

[30] Hernandez S, Tsuchiya Y, García–Ruiz JP, Lalioti V, Nielsen S, Cassio D, Sandoval IV. ATP7B copper-regulated traffic and association with the tight junctions: Copper excretion into the bile. Gastroenterology. 2008;134(1):1215–1223. 18395099 10.1053/j.gastro.2008.01.043

[31] Cen D, Brayton D, Shahandeh B, Meyskens FL Jr, Farmer PJ. Disulfiram facilitates intracellular Cu uptake and induces apoptosis in human melanoma cells. J Med Chem. 2004;47(27):6914–6920. 15615540 10.1021/jm049568z

[32] Kirshner JR, He S, Balasubramanyam V, Kepros J, Yang CY, Zhang M, du Z, Barsoum J, Bertin J. Elesclomol induces cancer cell apoptosis through oxidative stress. Mol Cancer Ther. 2008;7(8):2319–2327. 18723479 10.1158/1535-7163.MCT-08-0298

[33] Sabharwal SS, Schumacker PT. Mitochondrial ROS in cancer: Initiators, amplifiers or an Achilles’ heel? Nat Rev Cancer. 2014;14(11):709–721. 25342630 10.1038/nrc3803 PMC4657553

[34] Wang Y, Chen Y, Zhang J, Yang Y, Fleishman JS, Wang Y, Wang J, Chen J, Li Y, Wang H. Cuproptosis: A novel therapeutic target for overcoming cancer drug resistance. Drug Resist Updat. 2024;72: Article 101018. 37979442 10.1016/j.drup.2023.101018

[35] Zhang Z, Zeng X, Wu Y, Liu Y, Zhang X, Song Z. Cuproptosis-related risk score predicts prognosis and characterizes the tumor microenvironment in hepatocellular carcinoma. Front Immunol. 2022;13:925618. 35898502 10.3389/fimmu.2022.925618 PMC9311491

[36] Yang L, Zhang Y, Wang Y, Jiang P, Liu F, Feng N. Ferredoxin 1 is a cuproptosis-key gene responsible for tumor immunity and drug sensitivity: A pan-cancer analysis. Front Pharmacol. 2022;13:938134. 36210836 10.3389/fphar.2022.938134 PMC9532935

[37] Fang Y, Tian S, Pan Y, Li W, Wang Q, Tang Y, Yu T, Wu X, Shi Y, Ma P, et al. Pyroptosis: A new frontier in cancer. Biomed Pharmacother. 2020;121: Article 109595. 31710896 10.1016/j.biopha.2019.109595

[38] Tang X, Ren X, Huang T, Miao Y, Ha W, Li Z, Yang L, Mi D. Prognostic and immunological significance of the molecular subtypes and risk signatures based on cuproptosis in hepatocellular carcinoma. Mediat Inflamm. 2023;2023:3951940. 10.1155/2023/3951940 PMC10139815 37124062

[39] Li Y, Zeng X. A novel cuproptosis-related prognostic gene signature and validation of differential expression in hepatocellular carcinoma. Front Pharmacol. 2023;13:1081952. 36703728 10.3389/fphar.2022.1081952 PMC9871247

[40] Petruzzelli R, Polishchuk RS. Activity and trafficking of copper-transporting ATPases in tumor development and defense against platinum-based drugs. Cells. 2019;8(9):1080. 31540259 10.3390/cells8091080 PMC6769697

[41] Xue Q, Kang R, Klionsky DJ, Tang D, Liu J, Chen X. Copper metabolism in cell death and autophagy. Autophagy. 2023;19(8):2175–2195. 37055935 10.1080/15548627.2023.2200554 PMC10351475

[42] Chen J, Jiang Y, Shi H, Peng Y, Fan X, Li C. The molecular mechanisms of copper metabolism and its roles in human diseases. Pflugers Arch - Eur J Physiol. 2020;472(10):1415–1429. 32506322 10.1007/s00424-020-02412-2

[43] Zhang B, Burke R. Copper homeostasis and the ubiquitin proteasome system. Metallomics. 2023;15(3):mfad010. 36822629 10.1093/mtomcs/mfad010 PMC10022722

[44] Tadini-Buoninsegni F, Smeazzetto S. Mechanisms of charge transfer in human copper ATPases ATP7A and ATP7B. IUBMB Life. 2017;69(4):218–225. 28164426 10.1002/iub.1603

[45] Zhang H, Shi Y, Yi Q, Wang C, Xia Q, Zhang Y, Jiang W, Qi J. A novel defined cuproptosis-related gene signature for predicting the prognosis of lung adenocarcinoma. Front Genet. 2022;13:975185. 36046242 10.3389/fgene.2022.975185 PMC9421257

[46] Yagi T, Sawada K, Miyamoto M, Shimizu A, Oi Y, Toda A, Nakamura K, Kinose Y, Kodama M, Hashimoto K, et al. Continuous administration of anti-VEGFA antibody upregulates PAI-1 secretion from ovarian cancer cells via miR-143-3p downregulation. Mol Cancer Res. 2023;21(10):1093–1106. 37327051 10.1158/1541-7786.MCR-23-0015

[47] Nie H, Wang H, Zhang M, Ning Y, Chen X, Zhang Z, Hu X, Zhao Q, Chen P, Fang J, et al. Comprehensive analysis of cuproptosis-related genes in prognosis, tumor microenvironment infiltration, and immunotherapy response in gastric cancer. J Cancer Res Clin Oncol. 2022;149(8):5453–5468. 36462036 10.1007/s00432-022-04474-4 PMC11797702

[48] Huang X, Wang T, Ye J, Feng H, Zhang X, Ma X, Wang B, Huang Y, Zhang X. FDX1 expression predicts favourable prognosis in clear cell renal cell carcinoma identified by bioinformatics and tissue microarray analysis. Front Genet. 2022;13: Article 994741. 36186457 10.3389/fgene.2022.994741 PMC9523472

[49] Wang L, Cao Y, Guo W, Xu J. High expression of cuproptosis-related gene FDX1 in relation to good prognosis and immune cells infiltration in colon adenocarcinoma (COAD). J Cancer Res Clin Oncol. 2023;149(1):15–24. 36173462 10.1007/s00432-022-04382-7 PMC9889456

[50] Wu G, Peng H, Tang M, Yang M, Wang J, Hu Y, Li Z, Li J, Li Z, Song L. ZNF711 down-regulation promotes CISPLATIN resistance in epithelial ovarian cancer via interacting with JHDM2A and suppressing SLC31A1 expression. EBioMedicine. 2021;71: Article 103558. 34521054 10.1016/j.ebiom.2021.103558 PMC8441092

[51] Mangala LS, Zuzel V, Schmandt R, Leshane ES, Halder JB, Armaiz-Pena GN, Spannuth WA, Tanaka T, Shahzad MMK, Lin YG, et al. Therapeutic targeting of ATP7B in ovarian carcinoma. Clin Cancer Res. 2009;15(11):3770–3780. 19470734 10.1158/1078-0432.CCR-08-2306 PMC2752981

[52] Samimi G, Safaei R, Katano K, Holzer AK, Rochdi M, Tomioka M, Goodman M, Howell SB. Increased expression of the copper efflux transporter ATP7A mediates resistance to cisplatin, carboplatin, and oxaliplatin in ovarian cancer cells. Clin Cancer Res. 2004;10(14):4661–4669. 15269138 10.1158/1078-0432.CCR-04-0137

[53] Grivennikov SI, Greten FR, Karin M. Immunity, inflammation, and cancer. Cell. 2010;140(6):883–899. 20303878 10.1016/j.cell.2010.01.025 PMC2866629

[54] Del Prete A, Schioppa T, Tiberio L, Stabile H, Sozzani S. Leukocyte trafficking in tumor microenvironment. Curr Opin Pharmacol. 2017;35:40–47. 28577499 10.1016/j.coph.2017.05.004

[55] Pottier C, Wheatherspoon A, Roncarati P, Longuespée R, Herfs M, Duray A, Delvenne P, Quatresooz P. The importance of the tumor microenvironment in the therapeutic management of cancer. Expert Rev Anticancer Ther. 2015;15(8):943–954. 26098949 10.1586/14737140.2015.1059279

[56] Arneth B. Tumor microenvironment. Medicina (Kaunas). 2019;59(1):15. 10.3390/medicina56010015 PMC7023392 31906017

[57] Janeway CA Jr. Approaching the asymptote? Evolution and revolution in immunology. Cold Spring Harb Symp Quant Biol. 1989;54(Part 1):1–13. 10.1101/sqb.1989.054.01.003 2700931

[58] Hu F, Huang J, Bing T, Mou W, Li D, Zhang H, Chen Y, Jin Q, Yu Y, Yang Z. Stimulus-responsive copper complex nanoparticles induce cuproptosis for augmented cancer immunotherapy. Adv Sci. 2024;11:e2309388. 10.1002/advs.202309388 PMC10987162 38269649

[59] Ma M, Jiang W, Zhou R. DAMPs and DAMP-sensing receptors in inflammation and diseases. Immunity. 2024;57(4):752–771. 38599169 10.1016/j.immuni.2024.03.002

[60] Cheng F, Peng G, Lu Y, Wang K, Ju Q, Ju Y, Ouyang M. Relationship between copper and immunity: The potential role of copper in tumor immunity. Front Oncol. 2022;12:1019153. 36419894 10.3389/fonc.2022.1019153 PMC9676660

[61] Feig C, Jones JO, Kraman M, Wells RJB, Deonarine A, Chan DS, Connell CM, Roberts EW, Zhao Q, Caballero OL, et al. Targeting CXCL12 from FAP-expressing carcinoma-associated fibroblasts synergizes with anti-PD-L1 immunotherapy in pancreatic cancer. Proc Natl Acad Sci U S A. 2013;110:20212–20217. 24277834 10.1073/pnas.1320318110 PMC3864274

[62] Lu S, Li Y, Yu Y. Glutathione-scavenging celastrol-Cu nanoparticles induce self-amplified cuproptosis for augmented cancer immunotherapy. Adv Mater. 2024;36(35):e2404971. 38935977 10.1002/adma.202404971

[63] Zhao R, Sukocheva O, Tse E, Neganova M, Aleksandrova Y, Zheng Y, Gu H, Zhao D, Madhunapantula SRV, Zhu X, et al. Cuproptosis, the novel type of oxidation-induced cell death in thoracic cancers: Can it enhance the success of immunotherapy? Cell Commun Signal. 2024;22(1):379. 39068453 10.1186/s12964-024-01743-2 PMC11282696

[64] Turner JR. Intestinal mucosal barrier function in health and disease. Nat Rev Immunol. 2009;9(11):799–809. 19855405 10.1038/nri2653

[65] Cai R, Cheng C, Chen J, Xu X, Ding C, Gu B. Interactions of commensal and pathogenic microorganisms with the mucus layer in the colon. Gut Microbes. 2020;11(4):680–690. 32223365 10.1080/19490976.2020.1735606 PMC7524288

[66] Wu LM, Sankaran SJ, Plank LD, Windsor JA, Petrov MS. Meta-analysis of gut barrier dysfunction in patients with acute pancreatitis. Br J Surg. 2014;101(13):1644–1656. 25334028 10.1002/bjs.9665

[67] Puppa MJ, White JP, Sato S, Cairns M, Baynes JW, Carson JA. Gut barrier dysfunction in the Apc^Min/+^ mouse model of colon cancer cachexia. Biochim Biophys Acta. 2011;1812(12):1601–1606. 21914473 10.1016/j.bbadis.2011.08.010 PMC3205242

[68] Macpherson AJ, Uhr T. Induction of protective IgA by intestinal dendritic cells carrying commensal bacteria. Science. 2004;303(5664):1662–1665. 15016999 10.1126/science.1091334

[69] Hooper LV, Littman DR, Macpherson AJ. Interactions between the microbiota and the immune system. Science. 2012;336(6086):1268–1273. 22674334 10.1126/science.1223490 PMC4420145

[70] Stockinger B, Shah K, Wincent E. AHR in the intestinal microenvironment: Safeguarding barrier function. Nat Rev Gastroenterol Hepatol. 2021;18(8):559–570. 33742166 10.1038/s41575-021-00430-8 PMC7611426

[71] Dolcetti R, De Re V, Canzonieri V. Immunotherapy for gastric cancer: Time for a personalized approach? Int J Mol Sci. 2018;19(6):1602. 29844297 10.3390/ijms19061602 PMC6032163

[72] Li A, Yi M, Qin S, Song Y, Chu Q, Wu K. Activating cGAS-STING pathway for the optimal effect of cancer immunotherapy. J Hematol Oncol. 2019;12(1):35. 30935414 10.1186/s13045-019-0721-x PMC6444510

[73] Zheng J, Mo J, Zhu T, Zhuo W, Yi Y, Hu S, Yin J, Zhang W, Zhou H, Liu Z. Comprehensive elaboration of the cGAS-STING signaling axis in cancer development and immunotherapy. Mol Cancer. 2020;19(1):133. 32854711 10.1186/s12943-020-01250-1 PMC7450153

[74] Liu WQ, Lin WR, Yan L, Xu WH, Yang J. Copper homeostasis and cuproptosis in cancer immunity and therapy. Immunol Rev. 2023;321(1):211–227. 37715546 10.1111/imr.13276

[75] Théry C, Amigorena S. The cell biology of antigen presentation in dendritic cells. Curr Opin Immunol. 2001;13(1):45–51. 11154916 10.1016/s0952-7915(00)00180-1

[76] Gupta G, Cappellini F, Farcal L, Gornati R, Bernardini G, Fadeel B. Copper oxide nanoparticles trigger macrophage cell death with misfolding of Cu/Zn superoxide dismutase 1 (SOD1). Part Fibre Toxicol. 2022;19(1):33. 35538581 10.1186/s12989-022-00467-w PMC9088059

[77] Zheng Z, Zhang J, Jiang J, He Y, Zhang W, Mo X, Kang X, Xu Q, Wang B, Huang Y. Remodeling tumor immune microenvironment (TIME) for glioma therapy using multi-targeting liposomal codelivery. J Immunother Cancer. 2020;8(2):e000207. 32817393 10.1136/jitc-2019-000207 PMC7437977

[78] Gao X, Huang H, Pan C, Mei Z, Yin S, Zhou L, Zheng S. Disulfiram/copper induces immunogenic cell death and enhances CD47 blockade in hepatocellular carcinoma. Cancers (Basel). 2022;14(19):4715. 36230638 10.3390/cancers14194715 PMC9564202

[79] Chen Y, Cui W, Li X, Yang H. Interaction between commensal bacteria, immune response and the intestinal barrier in inflammatory bowel disease. Front Immunol. 2021;12: Article 761981. 34858414 10.3389/fimmu.2021.761981 PMC8632219

[80] Na YR, Stakenborg M, Seok SH, Matteoli G. Macrophages in intestinal inflammation and resolution: A potential therapeutic target in IBD. Nat Rev Gastroenterol Hepatol. 2019;16(9):531–543. 31312042 10.1038/s41575-019-0172-4

[81] Kahlson MA, Dixon SJ. Copper-induced cell death. Science. 2022;375(6586):1231–1232. 35298241 10.1126/science.abo3959

[82] Iborra M, Moret I, Rausell F, Bastida G, Aguas M, Cerrillo E, Nos P, Beltrán B. Role of oxidative stress and antioxidant enzymes in Crohn’s disease. Biochem Soc Trans. 2011;39(4):1102–1106. 21787356 10.1042/BST0391102

[83] Sahoo DK, Heilmann RM, Paital B, Patel A, Yadav VK, Wong D, Jergens AE. Oxidative stress, hormones, and effects of natural antioxidants on intestinal inflammation in inflammatory bowel disease. Front Endocrinol (Lausanne). 2023;14:1217165. 37701897 10.3389/fendo.2023.1217165 PMC10493311

[84] Pérez S, Taléns-Visconti R, Rius-Pérez S, Finamor I, Sastre J. Redox signaling in the gastrointestinal tract. Free Radic Biol Med. 2017;104:75–103. 28062361 10.1016/j.freeradbiomed.2016.12.048

[85] Colgan SP, Taylor CT. Hypoxia: An alarm signal during intestinal inflammation. Nat Rev Gastroenterol Hepatol. 2010;7(5):281–287. 20368740 10.1038/nrgastro.2010.39 PMC4077542

[86] Jian Z, Guo H, Liu H, Cui H, Fang J, Zuo Z, Deng J, Li Y, Wang X, Zhao L. Oxidative stress, apoptosis and inflammatory responses involved in copper-induced pulmonary toxicity in mice. Aging. 2020;12(17):16867–16886. 32952128 10.18632/aging.103585 PMC7521514

[87] Gaetke LM, Chow CK. Copper toxicity, oxidative stress, and antioxidant nutrients. Toxicology. 2003;189(1–2):147–163. 12821289 10.1016/s0300-483x(03)00159-8

[88] Yip NC, Fombon IS, Liu P, Brown S, Kannappan V, Armesilla AL, Xu B, Cassidy J, Darling JL, Wang W. Disulfiram modulated ROS–MAPK and NFκB pathways and targeted breast cancer cells with cancer stem cell-like properties. Br J Cancer. 2011;104(10):1564–1574. 21487404 10.1038/bjc.2011.126 PMC3101904

[89] Turski ML, Brady DC, Kim HJ, Kim BE, Nose Y, Counter CM, Winge DR, Thiele DJ. A novel role for copper in Ras/mitogen-activated protein kinase signaling. Mol Cell Biol. 2012;32(7):1284–1295. 22290441 10.1128/MCB.05722-11 PMC3302449

[90] Rath E, Moschetta A, Haller D. Mitochondrial function—Gatekeeper of intestinal epithelial cell homeostasis. Nat Rev Gastroenterol Hepatol. 2018;15(8):497–516. 29844587 10.1038/s41575-018-0021-x

[91] Ho GT, Aird RE, Liu B, Boyapati RK, Kennedy NA, Dorward DA, Noble CL, Shimizu T, Carter RN, Chew ETS, et al. MDR1 deficiency impairs mitochondrial homeostasis and promotes intestinal inflammation. Mucosal Immunol. 2018;11(1):120–130. 28401939 10.1038/mi.2017.31 PMC5510721

[92] Jackson DN, Panopoulos M, Neumann WL, Turner K, Cantarel BL, Thompson-Snipes LA, Dassopoulos T, Feagins LA, Souza RF, Mills JC, et al. Mitochondrial dysfunction during loss of prohibitin 1 triggers Paneth cell defects and ileitis. Gut. 2020;69(11):1928–1938. 32111635 10.1136/gutjnl-2019-319523 PMC7483170

[93] Sünderhauf A, Hicken M, Schlichting H, Skibbe K, Ragab M, Raschdorf A, Hirose M, Schäffler H, Bokemeyer A, Bettenworth D, et al. Loss of mucosal p32/gC1qR/HABP1 triggers energy deficiency and impairs goblet cell differentiation in ulcerative colitis. Cell Mol Gastroenterol Hepatol. 2021;12(1):229–250. 33515804 10.1016/j.jcmgh.2021.01.017 PMC8135049

[94] Halliwell B, Gutteridge JMC, Halliwell B, Gutteridge JMC. Antioxidant defences synthesized in vivo. UK: Oxford University Press; 2015.

[95] Candellone A, Cerquetella M, Girolami F, Badino P, Odore R. Acute diarrhea in dogs: Current management and potential role of dietary polyphenols supplementation. Antioxidants. 2020;9(8):725. 32784917 10.3390/antiox9080725 PMC7465157

[96] Sahoo DK, Chainy GBN. Hormone-linked redox status and its modulation by antioxidants. Netherlands: Elsevier; 2023. 10.1016/bs.vh.2022.10.007 36707135

[97] Chainy GBN, Sahoo DK. Hormones and oxidative stress: An overview. Free Radic Res. 2019;54(1):1–26. 31868060 10.1080/10715762.2019.1702656

[98] Torres J, Mehandru S, Colombel JF, Peyrin-Biroulet L. Crohn’s disease. Lancet. 2017;389(10080):1741–1755. 27914655 10.1016/S0140-6736(16)31711-1

[99] Choy MC, Visvanathan K, De Cruz P. An overview of the innate and adaptive immune system in inflammatory bowel disease. Inflamm Bowel Dis. 2017;23(1):2–13. 27779499 10.1097/MIB.0000000000000955

[100] de Souza HSP, Fiocchi C, Iliopoulos D. The IBD interactome: An integrated view of aetiology, pathogenesis and therapy. Nat Rev Gastroenterol Hepatol. 2017;14(12):739–749. 28831186 10.1038/nrgastro.2017.110

[101] Leung CY, Weitz JS. Not by (good) microbes alone: Towards immunocommensal therapies. Trends Microbiol. 2019;27(4):294–302. 30655037 10.1016/j.tim.2018.12.006

[102] Solier S, Müller S, Cañeque T, Versini A, Mansart A, Sindikubwabo F, Baron L, Emam L, Gestraud P, Pantoș GD, et al. A druggable copper-signalling pathway that drives inflammation. Nature. 2023;617(7960):386–394. 37100912 10.1038/s41586-023-06017-4 PMC10131557

[103] Sun R, Chen L. Assessment of heavy metal pollution in topsoil around Beijing Metropolis. PLOS ONE. 2016;11(5): Article e0155350. 27159454 10.1371/journal.pone.0155350 PMC4861295

[104] Dai J, Yang X, Yuan Y, Jia Y, Liu G, Lin N, Xiao H, Zhang L, Chen J. Toxicity, gut microbiota and metabolome effects after copper exposure during early life in SD rats. Toxicology. 2020;433–434: Article 152395. 10.1016/j.tox.2020.152395 32027963

[105] Liao J, Li Q, Lei C, Yu W, Deng J, Guo J, Han Q, Hu L, Li Y, Pan J, et al. Toxic effects of copper on the jejunum and colon of pigs: Mechanisms related to gut barrier dysfunction and inflammation influenced by the gut microbiota. Food Funct. 2021;12:9642–9657. 34664585 10.1039/d1fo01286j

[106] Flemming A. Copper boosts pro-inflammatory state of macrophages. Nat Rev Immunol. 2023;23(6):344. 10.1038/s41577-023-00889-3 PMC10176277 37173540

[107] Liu T, Zhang L, Joo D, Sun SC. NF-κB signaling in inflammation. Signal Transduct Target Ther. 2017;2:17023. 29158945 10.1038/sigtrans.2017.23 PMC5661633

[108] Qiu W, Wu B, Wang X, Buchanan ME, Regueiro MD, Hartman DJ, Schoen RE, Yu J, Zhang L. PUMA-mediated intestinal epithelial apoptosis contributes to ulcerative colitis in humans and mice. J Clin Invest. 2011;121(5):1722–1732. 21490394 10.1172/JCI42917 PMC3083802

[109] Klomp AE, van de Sluis B, Klomp LW, Wijmenga C. The ubiquitously expressed MURR1 protein is absent in canine copper toxicosis. J Hepatol. 2003;39(5):703–709. 14568250 10.1016/s0168-8278(03)00380-5

[110] Maine GN, Burstein E. COMMD proteins: COMMing to the scene. Cell Mol Life Sci. 2007;64(15):1997–2005. 17497243 10.1007/s00018-007-7078-y PMC2938186

[111] van de Sluis B, Groot AJ, Wijmenga C, Vooijs M, Klomp LW. COMMD1: A novel protein involved in the proteolysis of proteins. Cell Cycle. 2007;6(17):2091–2098. 17786049 10.4161/cc.6.17.4646

[112] Maine GN, Mao X, Komarck CM, Burstein E. COMMD1 promotes the ubiquitination of NF-κB subunits through a cullin-containing ubiquitin ligase. EMBO J. 2007;26(2):436–447. 17183367 10.1038/sj.emboj.7601489 PMC1783443

[113] Burstein E, Hoberg JE, Wilkinson AS, Rumble JM, Csomos RA, Komarck CM, Maine GN, Wilkinson JC, Mayo MW, Duckett CS. COMMD proteins, a novel family of structural and functional homologs of MURR1*. J Biol Chem. 2005;280(23):22222–22232. 15799966 10.1074/jbc.M501928200

[114] de Bie P, van de Sluis B, Burstein E, Duran KJ, Berger R, Duckett CS, Wijmenga C, Klomp LWJ. Characterization of COMMD protein–protein interactions in NF-κB signal ling. Biochem J. 398(1):63–71. 16573520 10.1042/BJ20051664 PMC1525016

[115] Geng H, Wittwer T, Dittrich-Breiholz O, Kracht M, Schmitz ML. Phosphorylation of NF-κB p65 at Ser468 controls its COMMD1-dependent ubiquitination and target gene-specific proteasomal elimination. EMBO Rep. 2009;10(4):381–386. 19270718 10.1038/embor.2009.10 PMC2672889

[116] de Bie P, van de Sluis B, Burstein E, Duran KJ, Berger R, Duckett CS, Wijmenga C, Klomp LWJ. Characterization of COMMD protein–protein interactions in NF-κB signalling. Biochem J. 2006;398(1):63–71. 16573520 10.1042/BJ20051664 PMC1525016

[117] Ganesh L, Burstein E, Guha-Niyogi A, Louder MK, Mascola JR, Klomp LWJ, Wijmenga C, Duckett CS, Nabel GJ. The gene product Murr1 restricts HIV-1 replication in resting CD4+ lymphocytes. Nature. 2003;426(6968):853–857. 14685242 10.1038/nature02171

[118] Li H, Chan L, Bartuzi P, Melton SD, Weber A, Ben–Shlomo S, Varol C, Raetz M, Mao X, Starokadomskyy P, et al. Copper metabolism domain-containing 1 represses genes that promote inflammation and protects mice from colitis and colitis-associated cancer. Gastroenterology. 2014;147(1):184–195 e183. 24727021 10.1053/j.gastro.2014.04.007 PMC4086320

[119] Zhu H, Li YR. Oxidative stress and redox signaling mechanisms of inflammatory bowel disease: Updated experimental and clinical evidence. Exp Biol Med (Maywood). 2012;237(5):474–480. 22442342 10.1258/ebm.2011.011358

[120] Pan Q, Bao LW, Merajver SD. Tetrathiomolybdate inhibits angiogenesis and metastasis through suppression of the NFkappaB signaling cascade. Mol Cancer Res. 2003;1(10):701–706. 12939395

[121] Xiao J, Guo X, Wang Z. Crosstalk between hypoxia-inducible factor-1α and short-chain fatty acids in inflammatory bowel disease: Key clues toward unraveling the mystery. Front Immunol. 2024;15:1385907. 38605960 10.3389/fimmu.2024.1385907 PMC11007100

[122] Dengler VL, Galbraith MD, Espinosa JM. Transcriptional regulation by hypoxia inducible factors. Crit Rev Biochem Mol Biol. 2013;49(1):1–15. 24099156 10.3109/10409238.2013.838205 PMC4342852

[123] Taylor CT, Colgan SP. Regulation of immunity and inflammation by hypoxia in immunological niches. Nat Rev Immunol. 2017;17(12):774–785. 28972206 10.1038/nri.2017.103 PMC5799081

[124] Watts ER, Walmsley SR. Inflammation and hypoxia: HIF and PHD isoform selectivity. Trends Mol Med. 2019;25(1):33–46. 30442494 10.1016/j.molmed.2018.10.006

[125] Martin F, Linden T, Katschinski D̈M, Oehme F, Flamme I, Mukhopadhyay CK, Eckhardt K, Tröger J, Barth S, Camenisch G, et al. Copper-dependent activation of hypoxia-inducible factor (HIF)-1: Implications for ceruloplasmin regulation. Blood. 2005;105(12):4613–4619. 15741220 10.1182/blood-2004-10-3980

[126] Feng W, Ye F, Xue W, Zhou Z, Kang YJ. Copper regulation of hypoxia-inducible factor-1 activity. Mol Pharmacol. 2009;75(1):174–182. 18842833 10.1124/mol.108.051516 PMC2685058

[127] Liu X, Zhang W, Wu Z, Yang Y, Kang YJ. Copper levels affect targeting of hypoxia-inducible factor 1alpha to the promoters of hypoxia-regulated genes. J Biol Chem. 2018;293(38):14669–14677. 30082314 10.1074/jbc.RA118.001764 PMC6153277

[128] Zhang Z, Qiu L, Lin C, Yang H, Fu H, Li R, Kang YJ. Copper-dependent and -independent hypoxia-inducible factor-1 regulation of gene expression. Metallomics. 2014;6(10):1889–1893. 25100165 10.1039/c4mt00052h

[129] Xie H, Kang YJ. Role of copper in angiogenesis and its medicinal implications. Curr Med Chem. 2009;16(10):1304–1314. 19355887 10.2174/092986709787846622

[130] Chen M, Huang Z, Xia M, Ding Y, Shan T, Guan Z, Dai X, Xu X, Huang Y, Huang M, et al. Glutathione-responsive copper-disulfiram nanoparticles for enhanced tumor chemotherapy. J Control Release. 2022;341:351–363. 34856225 10.1016/j.jconrel.2021.11.041

[131] Tang B, Zhu J, Liu F, Ding J, Wang Y, Fang S, zheng L, Qiu R, Chen M, Shu G, et al. xCT contributes to colorectal cancer tumorigenesis through upregulation of the MELK oncogene and activation of the AKT/mTOR cascade. Cell Death Dis. 2022;13(4):373. 35440604 10.1038/s41419-022-04827-4 PMC9019093

[132] Cao Y, Langer R, Ferrara N. Targeting angiogenesis in oncology, ophthalmology and beyond. Nat Rev Drug Discov. 2023;22(6):476–495. 37041221 10.1038/s41573-023-00671-z

[133] Jiménez-Valerio G, Casanovas O. Angiogenesis and metabolism: Entwined for therapy resistance. Trends Cancer. 2017;3(1):10–18. 28718423 10.1016/j.trecan.2016.11.007

[134] McAuslan BR, Reilly W. Endothelial cell phagokinesis in response to specific metal ions. Exp Cell Res. 1980;130(1):147–157. 6161014 10.1016/0014-4827(80)90051-8

[135] Sivaraja V, Kumar TKS, Rajalingam D, Graziani I, Prudovsky I, Yu C. Copper binding affinity of S100A13, a key component of the FGF-1 nonclassical copper-dependent release complex. Biophys J. 2006;91(5):1832–1843. 16766622 10.1529/biophysj.105.079988 PMC1544301

[136] Mandinov L, Mandinova A, Kyurkchiev S, Kyurkchiev D, Kehayov I, Kolev V, Soldi R, Bagala C, de Muinck ED, Lindner V, et al. Copper chelation represses the vascular response to injury. Proc Natl Acad Sci USA. 2003;100(11):6700–6705. 12754378 10.1073/pnas.1231994100 PMC164510

[137] Ash D, Sudhahar V, Youn SW, Okur MN, das A, O’Bryan JP, McMenamin M, Hou Y, Kaplan JH, Fukai T, et al. The P-type ATPase transporter ATP7A promotes angiogenesis by limiting autophagic degradation of VEGFR2. Nat Commun. 2021;12(1):3091. 34035268 10.1038/s41467-021-23408-1 PMC8149886

[138] Kohno T, Urao N, Ashino T, Sudhahar V, McKinney RD, Hamakubo T, Iwanari H, Ushio-Fukai M, Fukai T. Novel role of copper transport protein antioxidant-1 in neointimal formation after vascular injury. Arterioscler Thromb Vasc Biol. 2013;33(4):805–813. 23349186 10.1161/ATVBAHA.112.300862 PMC3600157

[139] Lowndes S, Harris AL. Copper chelation as an antiangiogenic therapy. Oncol Res. 2009;14(11–12):529–539. 10.3727/0965040042707952 15666995

[140] Zhang M, Chen L, Wang J. Recent advances in copper chaperone and cancer. Sci Sin Chim. 2018;48(5):1385–1393.

[141] Barker HE, Cox TR, Erler JT. The rationale for targeting the LOX family in cancer. Nat Rev Cancer. 2012;12(8):540–552. 22810810 10.1038/nrc3319

[142] Behrend L, Henderson G, Zwacka RM. Reactive oxygen species in oncogenic transformation. Biochem Soc Trans. 2003;31(Part 6):1441–1444. 14641084 10.1042/bst0311441

[143] Meier B, Radeke HH, Selle S, Younes M, Sies H, Resch K, Habermehl GG. Human fibroblasts release reactive oxygen species in response to interleukin-1 or tumour necrosis factor-α. Biochem J. 1989;263(2):539–545. 2556998 10.1042/bj2630539 PMC1133461

[144] Lo YY, Cruz TF. Involvement of reactive oxygen species in cytokine and growth factor induction of c-fos expression in chondrocytes. J Biol Chem. 1995;270(20):11727–11730. 7744816 10.1074/jbc.270.20.11727

[145] Sundaresan M, Yu Z-X, Ferrans VJ, Irani K, Finkel T. Requirement for generation of H_2_O_2_ for platelet-derived growth factor signal transduction. Science. 1995;270(5243):296–299. 7569979 10.1126/science.270.5234.296

[146] Roy D, Sarkar S, Felty Q. Levels of IL-1 beta control stimulatory/inhibitory growth of cancer cells. Front Biosci. 2006;11:889–898. 16146780 10.2741/1845

[147] Baldari S, Di Rocco G, Toietta G. Current biomedical use of copper chelation therapy. Int J Mol Sci. 2020;21(3):1069. 32041110 10.3390/ijms21031069 PMC7037088

[148] Yoshino H, Yamada Y, Enokida H, Osako Y, Tsuruda M, Kuroshima K, Sakaguchi T, Sugita S, Tatarano S, Nakagawa M. Targeting NPL4 via drug repositioning using disulfiram for the treatment of clear cell renal cell carcinoma. PLOS ONE. 2020;15(7): Article e0236119. 32667929 10.1371/journal.pone.0236119 PMC7363112

[149] Rieber M. Cancer pro-oxidant therapy through copper redox cycling: Repurposing disulfiram and tetrathiomolybdate. Curr Pharm Des. 2020;26(35):4461–4466. 32600223 10.2174/1381612826666200628022113

[150] Voli F, Valli E, Lerra L, Kimpton K, Saletta F, Giorgi FM, Mercatelli D, Rouaen JRC, Shen S, Murray JE, et al. Intratumoral copper modulates PD-L1 expression and influences tumor immune evasion. Cancer Res. 2020;80(19):4129–4144. 32816860 10.1158/0008-5472.CAN-20-0471

[151] Zhou B, Guo L, Zhang B, Liu S, Zhang K, Yan J, Zhang W, Yu M, Chen Z, Xu Y, et al. Disulfiram combined with copper induces immunosuppression via PD-L1 stabilization in hepatocellular carcinoma. Am J Cancer Res. 2019;9(11):2442–2455. 31815045 PMC6895448

[152] Baldari S, Rocco GD, Heffern MC, Su TA, Toietta G. Effects of copper chelation on BRAF^V600E^ positive colon carcinoma cells. Cancers. 2019;11(5):659. 31083627 10.3390/cancers11050659 PMC6562624

[153] Cai Z, Wang S, Li J. Treatment of inflammatory bowel disease: A comprehensive review. Front Med. 2021;8:765474. 10.3389/fmed.2021.765474 PMC8720971 34988090

[154] Kim SM, Hwang IK, Yoo DY, Eum WS, Kim DW, Shin MJ, Ahn EH, Jo HS, Ryu EJ, Yong JI, et al. Tat-antioxidant 1 protects against stress-induced hippocampal HT-22 cells death and attenuate ischaemic insult in animal model. J Cell Mol Med. 2015;19(6):1333–1345. 25781353 10.1111/jcmm.12513 PMC4459847

[155] van De Sluis B, Rothuizen J, Pearson PL, van Oost BA, Wijmenga C. Identification of a new copper metabolism gene by positional cloning in a purebred dog population. Hum Mol Genet. 2002;11(2):165–173. 11809725 10.1093/hmg/11.2.165

[156] Li H, Burstein E. COMMD1 regulates inflammation and colitis-associated cancer progression. Onco Targets Ther. 2014;3(7): Article e947891. 10.4161/21624011.2014.947891 PMC4292209 25610735

[157] van de Sluis B, Mao X, Zhai Y, Groot AJ, Vermeulen JF, van der Wall E, van Diest PJ, Hofker MH, Wijmenga C, Klomp LW, et al. COMMD1 disrupts HIF-1α/β dimerization and inhibits human tumor cell invasion. J Clin Invest. 2010;120(6):2119–2130. 20458141 10.1172/JCI40583 PMC2877941

[158] Taylor S, Lobo AJ. Diagnosis and treatment of inflammatory bowel disease. Practitioner. 2016;260(1795):19–23. 28994554

[159] Ojuawo A, Keith L. The serum concentrations of zinc, copper and selenium in children with inflammatory bowel disease. Cent Afr J Med. 2002;48(9-10):116–119. 14562534

[160] Aboelella NS, Brandle C, Kim T, Ding ZC, Zhou G. Oxidative stress in the tumor microenvironment and its relevance to cancer immunotherapy. Cancers. 2021;13(5):986. 33673398 10.3390/cancers13050986 PMC7956301

[161] Heaton DN, George GN, Garrison G, Winge DR. The mitochondrial copper metalochaperone Cox17 exists as an oligomeric, polycopper complex. Biochemistry. 2001;40(3):743–751. 11170391 10.1021/bi002315x

[162] Prohaska JR. Role of copper transporters in copper homeostasis. Am J Clin Nutr. 2008;88(3):826S–829S. 18779302 10.1093/ajcn/88.3.826S PMC2799992

[163] Cobine PA, Pierrel F, Winge DR. Copper trafficking to the mitochondrion and assembly of copper metalloenzymes. Biochim Biophys Acta. 2006;1763(7):759–772. 16631971 10.1016/j.bbamcr.2006.03.002

[164] Banci L, Bertini I, Ciofi-Baffoni S, Hadjiloi T, Martinelli M, Palumaa P. Mitochondrial copper(I) transfer from Cox17 to Sco1 is coupled to electron transfer. Proc Natl Acad Sci USA. 2008;105(19):6803–6808. 18458339 10.1073/pnas.0800019105 PMC2383975

[165] Singh RP, Jeyaraju DV, Voisin V, Hurren R, Xu C, Hawley JR, Barghout SH, Khan DH, Gronda M, Wang X, et al. Disrupting mitochondrial copper distribution inhibits leukemic stem cell self-renewal. Cell Stem Cell. 2020;26(6):926–937.e10. 32416059 10.1016/j.stem.2020.04.010

[166] Lukanović D, Herzog M, Kobal B, Černe K. The contribution of copper efflux transporters ATP7A and ATP7B to chemoresistance and personalized medicine in ovarian cancer. Biomed Pharmacother. 2020;129: Article 110401. 32570116 10.1016/j.biopha.2020.110401

[167] Zhou J, Yu Q, Song J, Li S, Li XL, Kang B, Chen HY, Xu JJ. Photothermally triggered copper payload release for cuproptosis-promoted cancer synergistic therapy. Angew Chem Int Ed Engl. 2023;62(12): Article e202213922. 36585379 10.1002/anie.202213922

[168] Jiang PC, Fan J, Zhang CD, Bai MH, Sun QQ, Chen QP, Mao W, Tang BF, Lan HY, Zhou YY, et al. Unraveling colorectal cancer and Pan-cancer immune heterogeneity and synthetic therapy response using cuproptosis and hypoxia regulators by multi-omic analysis and experimental validation. Int J Biol Sci. 2023;19(11):3526–3543. 37496994 10.7150/ijbs.84781 PMC10367564

[169] Cheng Z, Li M, Dey R, Chen Y. Nanomaterials for cancer therapy: Current progress and perspectives. J Hematol Oncol. 2021;14(1):85. 34059100 10.1186/s13045-021-01096-0 PMC8165984

[170] Lu S, Tian H, Li B, Li L, Jiang H, Gao Y, Zheng L, Huang C, Zhou Y, du Z, et al. An ellagic acid coordinated copper-based nanoplatform for efficiently overcoming cancer chemoresistance by cuproptosis and synergistic inhibition of cancer cell stemness. Small. 2024;20(17): Article e2309215. 38044295 10.1002/smll.202309215

[171] Xia Y, Gu M, Wang J, Zhang X, Shen T, Shi X, Yuan WE. Tumor microenvironment-activated, immunomodulatory nanosheets loaded with copper(II) and 5-FU for synergistic chemodynamic therapy and chemotherapy. J Colloid Interface Sci. 2024;653(Pt A):137–147. 37713912 10.1016/j.jcis.2023.09.042

[172] He L, Xu F, Li Y, Jin H, Lo P-C. Cupric-ion-promoted fabrication of oxygen-replenishing nanotherapeutics for synergistic chemo and photodynamic therapy against tumor hypoxia. Acta Biomater. 2023;162:57–71. 36944404 10.1016/j.actbio.2023.03.020

[173] Bing J, Zhou B, Chen M, Shen Y, Zhou M, Lin H, Wu W, Shi J. Nanomedicine-enabled concurrent regulations of ROS generation and copper metabolism for sonodynamic-amplified tumor therapy. Biomaterials. 2025;318: Article 123137. 39884132 10.1016/j.biomaterials.2025.123137

[174] Pashootan P, Saadati F, Fahimi H, Rahmati M, Strippoli R, Zarrabi A, Cordani M, Moosavi MA. Metal-based nanoparticles in cancer therapy: Exploring photodynamic therapy and its interplay with regulated cell death pathways. Int J Pharm. 2024;649: Article 123622. 37989403 10.1016/j.ijpharm.2023.123622

[175] Dou L, Lu E, Tian D, Li F, Deng L, Zhang Y. Adrenomedullin induces cisplatin chemoresistance in ovarian cancer through reprogramming of glucose metabolism. J Transl Int Med. 2023;11(2):169–177. 37408575 10.2478/jtim-2023-0091 PMC10318923

[176] Passirani C, Vessières A, La Regina G, Link W, Silvestri R. Modulating undruggable targets to overcome cancer therapy resistance. Drug Resist Updat. 2022;60: Article 100788. 35168144 10.1016/j.drup.2021.100788

[177] Hendrych M, Říhová K, Adamová B, Hradil V, Stiborek M, Vlček P, Hermanová M, Vašíčková J, Beneš P, Šmarda J, et al. Disulfiram increases the efficacy of 5-fluorouracil in organotypic cultures of colorectal carcinoma. Biomed Pharmacother. 2022;153: Article 113465. 36076577 10.1016/j.biopha.2022.113465

[178] Wangpaichitr M, Wu C, You M, Maher JC, Dinh V, Feun LG, Savaraj N. N’1,N’3-dimethyl-N’1,N’3-bis(phenylcarbonothioyl) propanedihydrazide (elesclomol) selectively kills cisplatin resistant lung cancer cells through reactive oxygen species (ROS). Cancers. 2009;1(1):23–38. 20535236 10.3390/cancers1010023 PMC2882109

[179] Buccarelli M, D’Alessandris QG, Matarrese P, Mollinari C, Signore M, Cappannini A, Martini M, D’Aliberti P, de Luca G, Pedini F, et al. Elesclomol-induced increase of mitochondrial reactive oxygen species impairs glioblastoma stem-like cell survival and tumor growth. J Exp Clin Cancer Res. 2021;40(1):228. 34253243 10.1186/s13046-021-02031-4 PMC8273992

[180] Wang X, Zhou M, Liu Y, Si Z. Cope with copper: From copper linked mechanisms to copper-based clinical cancer therapies. Cancer Lett. 2023;561: Article 216157. 37011869 10.1016/j.canlet.2023.216157

[181] Yang W, Wang Y, Huang Y, Yu J, Wang T, Li C, Yang L, Zhang P, Shi L, Yin Y, et al. 4-Octyl itaconate inhibits aerobic glycolysis by targeting GAPDH to promote cuproptosis in colorectal cancer. Biomed Pharmacother. 2023;159: Article 114301. 36706634 10.1016/j.biopha.2023.114301

[182] Wang H, Zhou X, Li C, Yan S, Feng C, He J, Li Z, Tu C. The emerging role of pyroptosis in pediatric cancers: From mechanism to therapy. J Hematol Oncol. 2022;15(1):140. 36209102 10.1186/s13045-022-01365-6 PMC9547461

[183] Fuchs Y, Steller H. Programmed cell death in animal development and disease. Cell. 2011;147(4):742–758. 22078876 10.1016/j.cell.2011.10.033 PMC4511103

[184] Kim BE, Nevitt T, Thiele DJ. Mechanisms for copper acquisition, distribution and regulation. Nat Chem Biol. 2008;4(3):176–185. 18277979 10.1038/nchembio.72

[185] Roy U, Gálvez EJC, Iljazovic A, Lesker TR, Błażejewski AJ, Pils MC, Heise U, Huber S, Flavell RA, Strowig T. Distinct microbial communities trigger colitis development upon intestinal barrier damage via innate or adaptive immune cells. Cell Rep. 2017;21(4):994–1008. 29069606 10.1016/j.celrep.2017.09.097 PMC5668567

[186] Zhang X, Tang B, Luo J, Yang Y, Weng Q, Fang S, Zhao Z, Tu J, Chen M, Ji J. Cuproptosis, ferroptosis and PANoptosis in tumor immune microenvironment remodeling and immunotherapy: Culprits or new hope. Mol Cancer. 2024;23(1):255. 39543600 10.1186/s12943-024-02130-8 PMC11566504

[187] Zhu Z, Zhao Q, Song W, Weng J, Li S, Guo T, Zhu C, Xu Y. A novel cuproptosis-related molecular pattern and its tumor microenvironment characterization in colorectal cancer. Front Immunol. 2022;13: Article 940774. 36248908 10.3389/fimmu.2022.940774 PMC9561547

[188] Liao J, Yang F, Bai Y, Yu W, Qiao N, Han Q, Zhang H, Guo J, Hu L, Li Y, et al. Metabolomics analysis reveals the effects of copper on mitochondria-mediated apoptosis in kidney of broiler chicken (*Gallus gallus*). J Inorg Biochem. 2021;224: Article 111581. 34419760 10.1016/j.jinorgbio.2021.111581

[189] Patwa J, Flora SJS. MiADMSA abrogates chronic copper-induced hepatic and immunological changes in Sprague Dawley rats. Food Chem Toxicol. 2020;145: Article 111692. 32871191 10.1016/j.fct.2020.111692

[190] Skalny AV, Sekacheva MI, Aschner M, Lobanova YN, Tinkov AA. Systemic essential metal and metalloid levels in patients with benign breast disease and breast cancer. Biol Trace Elem Res. 2022;200(12):5003–5012. 35048270 10.1007/s12011-022-03109-6

[191] Huang Y-L, Sheu J-Y, Lin T-H. Association between oxidative stress and changes of trace elements in patients with breast cancer. Clin Biochem. 1999;32(2):131–136. 10211630 10.1016/s0009-9120(98)00096-4

[192] Díez M, Arroyo M, Cerdàn FJ, Muñoz M, Martin MA, Balibrea JL. Serum and tissue trace metal levels in lung cancer. Oncology. 1989;46(4):230–234. 2740065 10.1159/000226722

[193] Habib FK, Dembinski TC, Stitch SR. The zinc and copper content of blood leucocytes and plasma from patients with benign and malignant prostates. Clin Chim Acta. 1980;104(3):329–335. 6156038 10.1016/0009-8981(80)90390-3

[194] Gupta SK, Shukla VK, Vaidya MP, Roy SK, Gupta S. Serum trace elements and Cu/Zn ratio in breast cancer patients. J Surg Oncol. 1991;46(3):178–181. 2011029 10.1002/jso.2930460311

[195] Sun L, Zhang Y, Yang B, Sun S, Zhang P, Luo Z, Feng T, Cui Z, Zhu T, Li Y, et al. Lactylation of METTL16 promotes cuproptosis via m(6)A-modification on FDX1 mRNA in gastric cancer. Nat Commun. 2023;14(1):6523. 37863889 10.1038/s41467-023-42025-8 PMC10589265

[196] Jia X, Tian J, Fu Y, Wang Y, Yang Y, Zhang M, Yang C, Liu Y. Identification of AURKA as a biomarker associated with cuproptosis and ferroptosis in HNSCC. Int J Mol Sci. 2024;25(8):4372. 38673957 10.3390/ijms25084372 PMC11050640

[197] Zhou Y, Xiao D, Jiang X, Nie C. EREG is the core onco-immunological biomarker of cuproptosis and mediates the cross-talk between VEGF and CD99 signaling in glioblastoma. J Transl Med. 2023;21(1):28. 36647156 10.1186/s12967-023-03883-4 PMC9843967

[198] Shu T, Wang X. Cuproptosis combines immune landscape providing prognostic biomarker in head and neck squamous carcinoma. Heliyon. 2023;9(5): Article e15494. 37215927 10.1016/j.heliyon.2023.e15494 PMC10196797

[199] Lu H, Zhou L, Zhang B, Xie Y, Yang H, Wang Z. Cuproptosis key gene FDX1 is a prognostic biomarker and associated with immune infiltration in glioma. Front Med (Lausanne). 2022;9: Article 939776. 36523779 10.3389/fmed.2022.939776 PMC9745336

[200] Lv H, Liu X, Zeng X, Liu Y, Zhang C, Zhang Q, Xu J. Comprehensive analysis of cuproptosis-related genes in immune infiltration and prognosis in melanoma. Front Pharmacol. 2022;13: Article 930041. 35837286 10.3389/fphar.2022.930041 PMC9273972

[201] Zhao X, Chen J, Yin S, Shi J, Zheng M, He C, Meng H, Han Y, Han J, Guo J, et al. The expression of cuproptosis-related genes in hepatocellular carcinoma and their relationships with prognosis. Front Oncol. 2022;12: Article 992468. 36313717 10.3389/fonc.2022.992468 PMC9614267

[202] Liu XS, Zeng J, Zhang YH, Zhang Y, Gao Y, Liu C, Pei ZJ. DARS2 is a prognostic biomarker and correlated with immune infiltrates and cuproptosis in lung adenocarcinoma. Am J Cancer Res. 2023;13(3):818–834. 37034224 PMC10077054

[203] Liu XS, Zhang Y, Liu ZY, Gao Y, Yuan LL, Zeng DB, Tan F, Wan HB, Pei ZJ. METTL3 as a novel diagnosis and treatment biomarker and its association with glycolysis, cuproptosis and ceRNA in oesophageal carcinoma. J Cell Mol Med. 2024;28(6): Article e18195. 38429907 10.1111/jcmm.18195 PMC10907846

[204] Xu L, Wu P, Rong A, Li K, Xiao X, Zhang Y, Wu H. Systematic pan-cancer analysis identifies cuproptosis-related gene DLAT as an immunological and prognostic biomarker. Aging (Albany NY). 2023;15(10):4269–4287. 37199628 10.18632/aging.204728 PMC10258010

[205] Zhang X, Zhou Y, Hu J, Yu X, Xu H, Ba Z, Zhang H, Sun Y, Wang R, du X, et al. Comprehensive analysis identifies cuproptosis-related gene DLAT as a potential prognostic and immunological biomarker in pancreatic adenocarcinoma. BMC Cancer. 2023;23(1):560. 37330494 10.1186/s12885-023-11042-7 PMC10276918

[206] Sun Z, Chen X, Huang X, Wu Y, Shao L, Zhou S, Zheng Z, Lin Y, Chen S. Cuproptosis and immune-related gene signature predicts immunotherapy response and prognosis in lung adenocarcinoma. Life. 2023;13(7):1583. 37511958 10.3390/life13071583 PMC10381686

[207] Li J, Yu T, Sun J, Zeng Z, Liu Z, Ma M, Zheng Z, He Y, Kang W. Comprehensive analysis of cuproptosis-related immune biomarker signature to enhance prognostic accuracy in gastric cancer. Aging (Albany NY). 2023;15(7):2772–2796. 37036489 10.18632/aging.204646 PMC10120894

[208] Cai Y, He Q, Liu W, Liang Q, Peng B, Li J, Zhang W, Kang F, Hong Q, Yan Y, et al. Comprehensive analysis of the potential cuproptosis-related biomarker LIAS that regulates prognosis and immunotherapy of pan-cancers. Front Oncol. 2022;12: Article 952129. 35982953 10.3389/fonc.2022.952129 PMC9379260

[209] Zhou B, Sha S, Wang Q, Sun S, Tao J, Zhu J, Dong L. The prognostic implications of cuproptosis-related gene signature and the potential of PPIC as a promising biomarker in cutaneous melanoma. Pigment Cell Melanoma Res. 2024;37(6):864–880. 39115044 10.1111/pcmr.13185

[210] Wang X, Chen D, Shi Y, Luo J, Zhang Y, Yuan X, Zhang C, Shu H, Yu W, Tian J. Copper and cuproptosis-related genes in hepatocellular carcinoma: Therapeutic biomarkers targeting tumor immune microenvironment and immune checkpoints. Front Immunol. 2023;14:1123231. 37153542 10.3389/fimmu.2023.1123231 PMC10157396

[211] Gu Y, Li C, Yan Y, Ming J, Li Y, Chao X, Wang T. Comprehensive analysis and verification of the prognostic significance of cuproptosis-related genes in colon adenocarcinoma. Int J Mol Sci. 2024;25(21):11830. 39519383 10.3390/ijms252111830 PMC11546850

[212] Fu H, Dong S, Li K. Identification of SLC31A1 as a prognostic biomarker and a target for therapeutics in breast cancer. Sci Rep. 2024;14(1):25120. 39448672 10.1038/s41598-024-76162-x PMC11502855

[213] Jin Y, Cai S, Zhou Y, Guo D, Zeng Y, Xu W, Sun Y, Shi Y, Xu Z, Liu Z, et al. Targeting SLC7A11/xCT improves radiofrequency ablation efficacy of HCC by dendritic cells mediated anti-tumor immune response. iMeta. 2024;3(6): Article e248. 39742309 10.1002/imt2.248 PMC11683471

[214] Bhat AA, Afzal M, Moglad E, Thapa R, Ali H, Almalki WH, Kazmi I, Alzarea SI, Gupta G, Subramaniyan V. lncRNAs as prognostic markers and therapeutic targets in cuproptosis-mediated cancer. Clin Exp Med. 2024;24:226. 39325172 10.1007/s10238-024-01491-0 PMC11427524

[215] Huang X, Hou Y, Weng X, Pang W, Hou L, Liang Y, Wang Y, Du L, Wu T, Yao M, et al. Diethyldithiocarbamate-copper complex (CuET) inhibits colorectal cancer progression via miR-16-5p and 15b-5p/ALDH1A3/PKM2 axis-mediated aerobic glycolysis pathway. Oncogenesis. 2021;10(1):4. 33419984 10.1038/s41389-020-00295-7 PMC7794448

[216] Gao W, Huang Z, Duan J, Nice EC, Lin J, Huang C. Elesclomol induces copper-dependent ferroptosis in colorectal cancer cells via degradation of ATP7A. Mol Oncol. 2021;15(12):3527–3544. 34390123 10.1002/1878-0261.13079 PMC8637554

[217] Wang W, McLeod HL, Cassidy J. Disulfiram-mediated inhibition of NF-kappaB activity enhances cytotoxicity of 5-fluorouracil in human colorectal cancer cell lines. Int J Cancer. 2003;104(4):504–511. 12584750 10.1002/ijc.10972

[218] Hu Y, Qian Y, Wei J, Jin T, Kong X, Cao H, Ding K. The disulfiram/copper complex induces autophagic cell death in colorectal cancer by targeting ULK1. Front Pharmacol. 2021;12:752825. 34887757 10.3389/fphar.2021.752825 PMC8650091

[219] You S-Y, Rui W, Chen ST, Chen HC, Liu XW, Huang J, Chen HY. Process of immunogenic cell death caused by disulfiram as the anti-colorectal cancer candidate. Biochem Biophys Res Commun. 2019;513(4):891–897. 31003768 10.1016/j.bbrc.2019.03.192

[220] Zuhra K, Panagaki T, Randi EB, Augsburger F, Blondel M, Friocourt G, Herault Y, Szabo C. Mechanism of cystathionine-β-synthase inhibition by disulfiram: The role of bis(N,N-diethyldithiocarbamate)-copper(II). Biochem Pharmacol. 2020;182: Article 114267. 33035509 10.1016/j.bcp.2020.114267

[221] Yang Y, Liang S, Geng H, Xiong M, Li M, Su Q, Jia F, Zhao Y, Wang K, Jiang J, et al. Proteomics revealed the crosstalk between copper stress and cuproptosis, and explored the feasibility of curcumin as anticancer copper ionophore. Free Radic Biol Med. 2022;193(Pt 2):638–647. 36395954 10.1016/j.freeradbiomed.2022.11.023

[222] Zhang W, Chen C, Shi H, Yang M, Liu Y, Ji P, Chen H, Tan RX, Li E. Curcumin is a biologically active copper chelator with antitumor activity. Phytomedicine. 2016;23(1):1–8. 26902401 10.1016/j.phymed.2015.11.005

[223] Barresi V, Spampinato G, Musso N, Trovato Salinaro A, Rizzarelli E, Condorelli DF. ATOX1 gene silencing increases susceptibility to anticancer therapy based on copper ionophores or chelating drugs. J Inorg Biochem. 2016;156:145–152. 26784148 10.1016/j.jinorgbio.2016.01.002

[224] Fatfat M, Merhi RA, Rahal O, Stoyanovsky DA, Zaki A, Haidar H, Kagan VE, Gali-Muhtasib H, Machaca K. Copper chelation selectively kills colon cancer cells through redox cycling and generation of reactive oxygen species. BMC Cancer. 2014;14:527. 25047035 10.1186/1471-2407-14-527 PMC4223620

[225] Shi X, Li Y, Jia M, Zhang Z, Huang L, Zhang M, Xun Q, Jiang D, Liu Y. A novel copper chelator for the suppression of colorectal cancer. Drug Dev Res. 2023;84(2):312–325. 36658741 10.1002/ddr.22034

[226] Rolim PM, Fidelis GP, Padilha CEA, Santos ES, Rocha HAO, Macedo GR. Phenolic profile and antioxidant activity from peels and seeds of melon (*Cucumis melo L. var. reticulatus*) and their antiproliferative effect in cancer cells. Braz J Med Biol Res. 2018;51(4): Article e6069. 29513789 10.1590/1414-431X20176069 PMC5856442

[227] Bormio Nunes JH, Hager S, Mathuber M, Pósa V, Roller A, Enyedy ÉA, Stefanelli A, Berger W, Keppler BK, Heffeter P, et al. Cancer cell resistance against the clinically investigated thiosemicarbazone COTI-2 is based on formation of intracellular copper complex glutathione adducts and ABCC1-mediated efflux. J Med Chem. 2020;63(22):13719–13732. 33190481 10.1021/acs.jmedchem.0c01277 PMC7706001

[228] Yu N, Zhu H, Yang Y, Tao Y, Tan F, Pei Q, Zhou Y, Song X, Tan Q, Pei H. Combination of Fe/Cu-chelators and docosahexaenoic acid: An exploration for the treatment of colorectal cancer. Oncotarget. 2017;8(31):51478–51491. 28881661 10.18632/oncotarget.17807 PMC5584262

[229] Gandin V, Ceresa C, Esposito G, Indraccolo S, Porchia M, Tisato F, Santini C, Pellei M, Marzano C. Therapeutic potential of the phosphino Cu(I) complex (HydroCuP) in the treatment of solid tumors. Sci Rep. 2017;7(1):13936. 29066771 10.1038/s41598-017-13698-1 PMC5655689

